# Supplementary material for: NRP1 Regulates CDC42 Activation to Promote Filopodia Formation in Endothelial Tip Cells
Source: Cell Rep. 2015 Jun 4;11(10):1577–90. doi: 10.1016/j.celrep.2015.05.018 (PMC4528263; doi:10.1016/j.celrep.2015.05.018)
Supplement: Document S1. Supplemental Experimental Procedures and Figures S1–S3 [file mmc1.pdf]

Cell Reports

Supplemental Information

NRP1 Regulates CDC42 Activation to Promote Filopodia Formation in Endothelial Tip Cells

Alessandro Fantin, Anastasia Lampropoulou, Gaia Gestri, Claudio Raimondi, Valentina Senatore, Ian Zachary, and Christiana Ruhrberg

Supplemental Information

Supplemental figures and legends:

Figure S1, related to Fig. 1. **Reduced NRP1 levels in heterozygous *Nrp1*-null mutant hindbrains.** (**A-D**) Non-saturated confocal z-stacks of E11.5 mouse hindbrains of the indicated genotypes, immunofluorescently labelled for IB4 and NRP1. (**B',D'**) The single NRP1 channels shown in (**B,D**) were converted into a heat map to illustrate reduced pixel intensity in heterozygous mutants, whereby areas of high intensity are shown in red and areas of low intensity are shown in (blue); arrowheads indicate examples of tip cells. Scale bar: 50 μm. (**E)** Quantitation of NRP1 pixel intensity in heterozygous mutants relative to wild type littermates.

Figure S2, related to Fig. 5**. Normal ISV sprouting in control MO- and *nrp1b* MO-treated zebrafish embryos and demonstration that neural apoptosis or proliferation defects are not responsible for ISV defects in *nrp1a/b* MO-treated zebrafish.** (**A**) Confocal z-stacks of trunks from MO-treated 32 hpf *Tg(fli1a:EGFP)*^{y5} zebrafish that were immunostained for GFP after apoptosis detection via TUNEL; embryos had been treated with control MO, *nrp1a/b* MO or *nrp1a/b* MO together with *tp53* MO, as indicated. The GFP single channels are shown in greyscale in the upper panels. The boxed areas are shown at higher magnification in the bottom panels. The squared bracket indicates the position of the neural tube. Note increased apoptosis in the neural tube of *nrp1a/b* MO-treated embryos, but not in fish treated with the *nrp1a/b* MO together with the *tp53* MO. (**B**) Confocal z-stacks of trunks from 32 hpf *Tg(kdrl:HsHRAS-mCherry)*^{s896} zebrafish immunostained for mCherry and pHH3 after treatment with control, *nrp1b* or *nrp1a/b* MO. The mCherry single channel is shown in greyscale in the upper panels. The boxed areas are shown at higher magnification in the bottom panels. Arrowheads indicate examples of proliferating endothelial cells in ISV sprouts. Scale bars: 100 μm.

Figure S3, related to Fig. 5. **Nrp1 knockdown reduces the migration speed of ISV sprouts.** Live imaging of *Tg(fli1a:EGFP)*^{y5} zebrafish embryos during ISV sprouting at 32 hpf. (**A**) Series of laser confocal projections extracted from time-lapse videos (supplemental Movie 1 and 2) shown for the trunk of a control (left side panels) and *nrp1a/b* MO-microinjected (right side panels) zebrafish embryo and visualised with Imaris normal shading volume rendering; scale bar: 25 μm. (**B**) Quantitation of the migration speed of single ISV sprouts; mean±SD, n≥5 sprouts; *P* value (*<0.05).

Supplemental movies and legends:

Movie S1, related for Figure 5. **Migration speed of ISV sprouts in control embryos.** Live imaging of *Tg(fli1a:EGFP)*^{y5} zebrafish embryo trunks during ISV sprouting at 32 hpf.

Movie S2, , related for Figure 5**. Migration speed of ISV sprouts after Nrp1 knockdown.** Live imaging of *Tg(fli1a:EGFP)*^{y5} zebrafish embryo trunks during ISV sprouting at 32 hpf after *nrp1a/b* MO-microinjection.

Supplemental Experimental Procedures

Mouse strains Animal procedures were performed in accordance with institutional and UK Home Office guidelines. Mice were mated in the evening and the morning of vaginal plug formation was counted as embryonic (E) day 0.5. We used mice carrying a null mutation for *Nrp1* on a CD1 background (Kitsukawa et al., 1997) to circumvent the embryonic lethality of homozygous mutants by E10.5 that is observed in the C57/Bl6 background (Jones et al., 2008). We also used $Nrp1^{Y297A}$ mice carrying a Y297A mutation that prevents VEGF-A binding to NRP1 (Fantin et al., 2014). For tamoxifen-induced, endothelial specific targeting of *Nrp1,* we used mice carrying floxed conditional *Nrp1* null alleles ($Nrp1^{fl/fl}$) together with *Pdgfb-iCre-ERT2-Egfp* with codon-improved *Cre* on a C57/Bl6 background (Fantin et al., 2013a). Tamoxifen (Sigma) was dissolved in peanut oil at 2 mg/ml, and 0.1 mg was administered on P2 and P3 via subcutaneous injections, followed by intraperitoneal injections on P4 and P5. ML141 (Sigma) was dissolved at 8 mM in DMSO and diluted to 4 mM in saline solution for injection of 0.08 mg into pups, whilst Imatinib (Cambridge Bioscience) was dissolved at 10 mM in water and diluted to 5 mM in saline solution for injection of 0.2 mg; both inhibitors were administered with a delivery regime similar to that used for tamoxifen treatment.

Wholemount immunolabelling, imaging and quantitative analysis of mouse hindbrains and retinas Mouse embryo hindbrains and postnatal retinas were immunolabelled as described (Fantin et al., 2013b; Pitulescu et al., 2010). To visualise blood vessels, we used biotinylated IB4 (cat. no. L2140, Sigma UK) followed by Alexa-conjugated streptavidin (Life Technologies). Samples were imaged with a LSM710 laser scanning confocal microscopes (Zeiss, Jena, Germany). For the 3-dimensional analysis of EC filopodia morphology with Imaris (BitPlane), IB4+ blood vessels and macrophages from high-resolution confocal z-stacks were masked, but filopodia extending from vessels were excluded from the mask; filopodia were automatically tracked with the Imaris Filament Tracer module. The endothelial tip cell number as well as filopodia number, length and thickness were determined in a minimum of two 0.05 mm^2 hindbrain regions, and the counts for each hindbrain averaged to yield a value for each hindbrain. In some experiments, rat anti-PECAM (cat. no. 553370, BD Bioscience) was detected with horseradish peroxidase (HRP)-conjugated rabbit anti-rat IgG (DAKO UK) and HRP-labelled samples were imaged with an MZ16 stereomicroscope (Leica) equipped with a ProgRes C14 camera (Jenoptics). The number of vascular branchpoints at E12.5 was determined in 3 randomly chosen 0.25 mm^2 regions in each hindbrain and then averaged. Tip cell density in the vascular front of the retina was quantified by normalising the number of filopodial bursts to the length of the vascular front, which was measured with Image J (http://imagej.nih.gov/ij/index.html). Vascular branchpoints in postnatal retinas were quantified using the Imaris Filament Tracer module in a minimum of three 0.16 mm^2 capillary areas behind the vascular front, and the counts for each retina averaged to yield a value for each retina. For all experiments, we calculated the mean of 3-12 independent samples. To evaluate NRP1 protein levels *in situ*, we combined IB4 staining with immunolabelling, using goat anti-rat NRP1 (cat. no. AF566, R&D Systems) followed by Cy3-conjugated rabbit anti-goat Fab fragment (Jackson Immuno) and acquired confocal z-stacks of the labelled tissue; we then rendered the single channel confocal z-stacks of NRP1 staining into heat-maps using the rainbow palette in Zeiss LSM Image Browser, with red, green and blue indicating high, medium and low pixel intensity, respectively. Images were processed with Photoshop CS4 (Adobe Inc.).

Gene expression analyses Hindbrain mRNA was extracted using TRI reagent (Sigma) and cDNA prepared using Superscript III reverse transcriptase (Invitrogen). For PCR amplification, we used SybrGreen (Applied Biosystems, USA) and the following oligonucleotide primers: *Nrp1* 5'-GAAGGTGAAATCGGAAAAGG-3' and 5'-GGTCTGTTGGTTTTGCACAG-3'; *Dll4* 5'-GCAGCTGTAAGGACCAGGAG-3' and 5'-ATTCGCAGGCATAACTGGAC-3'; *Ang2* 5'-CACAGCGAGCAGCTACAGTC-3' and 5'-ATAGCAACCGAGCTCTTGGA-3'; *Pecam* 5'-CGATGCGATGGTGTATAACG-3' and 5'-GTCACCTTGGGCTTGGATAC-3'; *Apln* 5'-GAGGAAATTTCGCAGACAGC-3' and 5'-GAGGAACTTGGTGGGTGAGA-3'; *Vegfr2* 5'-TCACCGAGAACAAGAACAAA-3' and 5'-TCCTATATCCTACAACCACAA-3'; *Actb* 5'-AAGGCCAACCGTGAAAAGAT-3' and 5'-GTGGTACGACCAGAGGCATAC-3'; *Hes1* 5'-TACCTGAAACACAGCAAAGC-3' and 5'-GGTAAACGAGCTTCATCTGC-3'; *Hey1* 5'-GGACGAGAATGGAAACTTGAG-3' and 5'-TTTCTAGCTTAGCAGATCCCT-3'; *Cdh5* 5'-GATGCAGATGACCCCACTGT-3' and 5'-AGGGCATCTTGTGTTTCCAC-3'. Amplicons were analysed with the quantitative 7500 Real-Time PCR System (Applied Biosystems) and SDS software v2.3 (Applied Biosystems) and DART-PCR software (Peirson et al., 2003).

Zebrafish experiments We injected *Tg(fli1a:EGFP)^{y5}* (Lawson and Weinstein, 2002) or *Tg(kdrl:HsHRAS-mCherry)^{s896}* (Chi et al., 2008) zebrafish embryos at the one cell stage with 0.6 pmol of the *nrp1a/b* translation blocking MO 5'-GAATCCTGGAGTTCGGAGTGCGGAA-3', previously published as a *nrp1a* MO (Hillman et al., 2011; Lee et al., 2002), 1.2 pmol of the *nrp1b* splicing blocking MO 5'-TATACCTGTACGGTGTATCTCATAG-3' (Wang et al., 2007) or 2.5 pmol of the standard control MO 5'-CCTCTTACCTCAGTTACAATTTATA-3' (Gene Tools). In some experiments, we co-injected 1 pmol of the translation blocking *tp53* MO 5'-GCGCCATTGCTTTGCAAGAATTG-3' (Chen et al., 2005) that inhibits apoptosis (e.g. Paridaen et al., 2011). Cell lysates from 32 hpf embryos were immunoblotted with a rabbit monoclonal antibody specific for the NRP1 cytoplasmic domain (cat no. 2621-1, Epitomics). Clustal Omega (https://www.ebi.ac.uk/Tools/msa/clustalo/) was used to align nucleotide and amino acid sequences of the two Nrp1 homologs with MO and antibody target sequences, respectively. In some experiments, embryos were treated with 25 or 75 µM ML141 or vehicle (DMSO) in the aquarium water from 8 to 32 hpf. To image vascular development, embryos were incubated at 28°C with phenylthiourea to prevent melanisation. 32 hpf embryos were then formaldehyde-fixed and immunostained with rabbit anti-GFP (cat. no. 598, MBL), chicken anti-GFP (cat. no. GFP-1020, Aves Labs) or rabbit anti-pHH3 (cat. no. 06-570, Millipore) and Alexa-conjugated goat anti-rabbit or anti-chicken antibodies (Life Technologies). Terminal deoxynucleotidyl transferase dUTP nick end labelling (TUNEL) was performed with the ApopTag kit according to the manufacturer's instructions (Millipore). 3-dimensional analysis of vessel sprouts and filopodia was carried out with Imaris as follows. Blood vessels and filopodia were tracked in high-resolution confocal z-stacks using Imaris Filament Tracer, while vessel sprout volume was measured using Imaris Surface rendering. We calculated the mean sprout values from 5-10 embryos per group in 2 independent experiments. For live imaging, 32 hpf embryos were anaesthetised in 0.01% tricaine and embedded in 1.5% low-melting point agarose. Time-lapse analysis was carried out on a Leica SPE confocal microscope for 4 hours by acquiring z-stacks of 15-20 confocal slices each every 10 minutes. The z-stacks were flattened by maximum projection and arranged in a time series using Imaris.

Cell culture and cell imaging HDMEC were cultured in MV2 media with supplements (Promocell, UK) and transfected with SMARTpool siRNA targeting NRP1 or ABL1 (Dharmacon, USA) or *Silencer*® negative control siRNA (Applied Biosystems, UK) using Lipofectamine RNAIMAX (Life Technologies). To evaluate the effect of NRP1 knockdown, HDMEC were serum-starved overnight, detached and plated on glass coverslips that had been coated overnight with 10 μg/ml FN (Sigma). In some experiments, untransfected cells were treated with vehicle (DMSO) or 7.5 μM ML141 before plating on FN; in other experiments, we additionally stimulated cells with 5 ng/ml VEGF165 for 15 min (R&D Systems). Stimulated cells were fixed, labelled with Alexa488-conjugated phalloidin and DAPI (Sigma) and then imaged on a Zeiss LSM700 confocal microscope.

CDC42 pull down assay, immunoprecipitation and immunoblotting To isolate GTP-bound CDC42, two different pull down assays were performed according to the manufacturer's instructions using glutathione agarose beads that were bound to either the p21-binding domain of PAK1 (Millipore) or the CDC42-binding domain of WASP (Cytoskeleton) via a GST tag. Immunoblotting of the precipitated proteins with an antibody specific for CDC42 (Millipore) identified GTP-bound CDC42. For this experiment, we serum-starved HDMEC overnight, detached and plated them on tissue culture plastic coated with 10 μg/ml FN for 30 minutes; in some experiments, we additionally stimulated for 15 min with 5 ng/ml VEGF165. In some experiments, HDMEC were transfected with siRNA targeting NRP1, ABL1 or control siRNA prior to plating on FN; in other experiments, cells were treated with vehicle or ML141, as described above. HDMEC were lysed according to the manufacturer's instructions and 300 μg protein was incubated with 10 μg beads at 4°C for 60 minutes. The bead supernatant was collected as the input control, while the bead pellet was washed and boiled for 5 minutes in 40 μl 2.5x Laemmli sample buffer to elute bound protein. CDC42 activation was calculated as the ratio between CDC42, detected by immunoblotting after pull down, and GST, detected by immunoblotting after pull down, or GAPDH, detected by immunoblotting in the input lysate (bead supernatant). For immunoprecipitation, HDMEC were lysed in 50 mM Tris pH 8.0 containing 50 mM KCl and 1% (v/v) Triton X-100 as well as protease inhibitor cocktail 2 and phosphatase inhibitor cocktail (Sigma) and incubated with goat anti-NRP1 (cat. no. sc-7239, Santa Cruz Biotechnology) or control goat IgG (Santa Cruz Biotechnology). For immunoblotting, heat-denatured samples were transferred to nitrocellulose membrane (Whatman, USA) after electrophoretic separation. We used the following antibodies for immunoblotting: mouse anti-CDC42 (cat. no. 17-441, Millipore), anti-GST (cat. no. G1160, Sigma), rabbit anti-NRP1 (cat. no. D62C6, Cell Signaling), anti-pCRKL (cat. no. 3181, Cell Signaling), and anti-GAPDH (cat. no. ab9485, Abcam) followed by appropriate HRP-conjugated secondary antibodies (Sigma).

Statistical analysis Error bars represent the standard deviation of the mean, unless otherwise stated in the figure legends. To determine if two data sets were significantly different, we calculated the *P*-value by performing a two-tailed unpaired Student's t-test; to compare more than two data sets, we additionally performed a one-way ANOVA followed by a Tukey post-hoc test; a P-value < 0.05 was considered significant. Statistical analyses were performed with Prism 5 (GraphPad Software) or Excel 12.2.6 (Microsoft Office).

Supplemental References

Chen, J., Ruan, H., Ng, S.M., Gao, C., Soo, H.M., Wu, W., Zhang, Z., Wen, Z., Lane, D.P., and Peng, J. (2005). Loss of function of def selectively up-regulates Delta113p53 expression to arrest expansion growth of digestive organs in zebrafish. Genes Dev *19*, 2900-2911.

Chi, N.C., Shaw, R.M., De Val, S., Kang, G., Jan, L.Y., Black, B.L., and Stainier, D.Y. (2008). Foxn4 directly regulates tbx2b expression and atrioventricular canal formation. Genes Dev *22*, 734-739.

Fantin, A., Herzog, B., Mahmoud, M., Yamaji, M., Plein, A., Denti, L., Ruhrberg, C., and Zachary, I. (2014). Neuropilin 1 (NRP1) hypomorphism combined with defective VEGF-A binding reveals novel roles for NRP1 in developmental and pathological angiogenesis. Development *141*, 556-562.

Fantin, A., Vieira, J.M., Plein, A., Denti, L., Fruttiger, M., Pollard, J.W., and Ruhrberg, C. (2013a). NRP1 acts cell autonomously in endothelium to promote tip cell function during sprouting angiogenesis. Blood *121*, 2352-2362.

Fantin, A., Vieira, J.M., Plein, A., Maden, C.H., and Ruhrberg, C. (2013b). The embryonic mouse hindbrain as a qualitative and quantitative model for studying the molecular and cellular mechanisms of angiogenesis. Nature Protocols *8*, 418-429.

Hillman, R.T., Feng, B.Y., Ni, J., Woo, W.M., Milenkovic, L., Hayden Gephart, M.G., Teruel, M.N., Oro, A.E., Chen, J.K., and Scott, M.P. (2011). Neuropilins are positive regulators of Hedgehog signal transduction. Genes & Development *25*, 2333-2346.

Jones, E.A., Yuan, L., Breant, C., Watts, R.J., and Eichmann, A. (2008). Separating genetic and hemodynamic defects in neuropilin 1 knockout embryos. Development *135*, 2479-2488.

Kitsukawa, T., Shimizu, M., Sanbo, M., Hirata, T., Taniguchi, M., Bekku, Y., Yagi, T., and Fujisawa, H. (1997). Neuropilin-semaphorin III/D-mediated chemorepulsive signals play a crucial role in peripheral nerve projection in mice. Neuron *19*, 995-1005.

Lawson, N.D., and Weinstein, B.M. (2002). In vivo imaging of embryonic vascular development using transgenic zebrafish. Dev Biol *248*, 307-318.

Lee, P., Goishi, K., Davidson, A.J., Mannix, R., Zon, L., and Klagsbrun, M. (2002). Neuropilin-1 is required for vascular development and is a mediator of VEGF-dependent angiogenesis in zebrafish. Proceedings of the National Academy of Sciences of the United States of America *99*, 10470-10475.

Paridaen, J.T., Janson, E., Utami, K.H., Pereboom, T.C., Essers, P.B., van Rooijen, C., Zivkovic, D., and MacInnes, A.W. (2011). The nucleolar GTP-binding proteins Gnl2 and nucleostemin are required for retinal neurogenesis in developing zebrafish. Dev Biol *355*, 286-301.

Peirson, S.N., Butler, J.N., and Foster, R.G. (2003). Experimental validation of novel and conventional approaches to quantitative real-time PCR data analysis. Nucleic Acids Res *31*, e73.

Pitulescu, M.E., Schmidt, I., Benedito, R., and Adams, R.H. (2010). Inducible gene targeting in the neonatal vasculature and analysis of retinal angiogenesis in mice. Nat Protoc *5*, 1518-1534.

Wang, L., Dutta, S.K., Kojima, T., Xu, X., Khosravi-Far, R., Ekker, S.C., and Mukhopadhyay, D. (2007). Neuropilin-1 modulates p53/caspases axis to promote endothelial cell survival. PLoS ONE *2*, e1161.

Figure S1

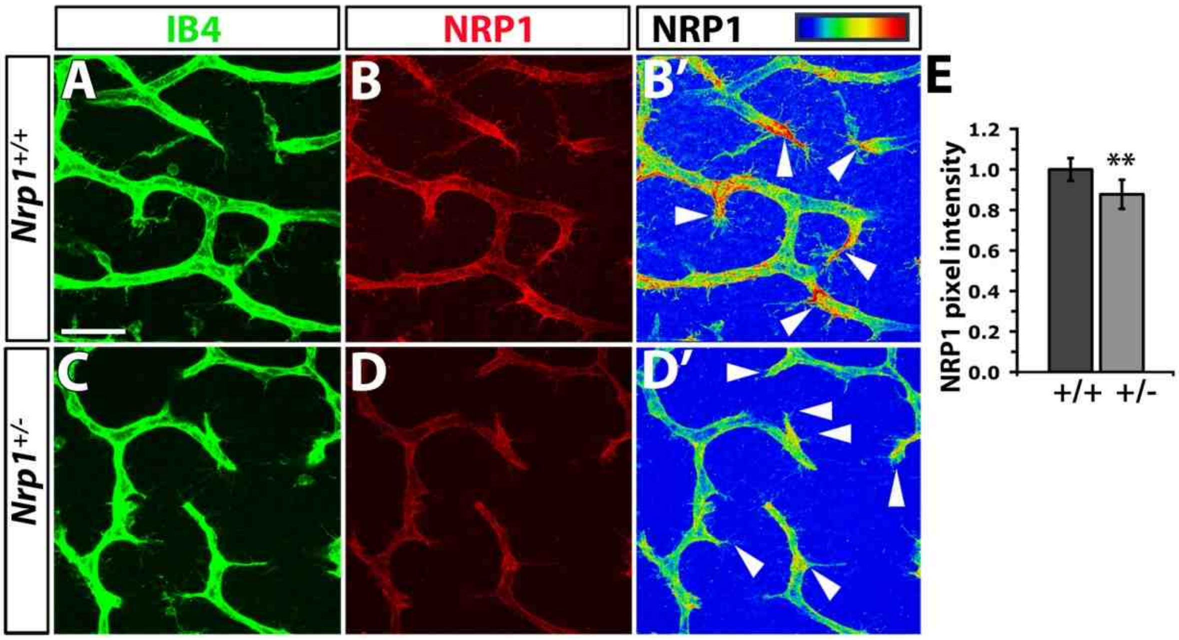
IB4
NRP1
NRP1
Nrp1+/+
Nrp1+/-
A
B
B'
C
D
D'
E
NRP1 pixel intensity
1.2
1.0
0.8
0.6
0.4
0.2
0.0
**
+/+
+/-

Figure S2

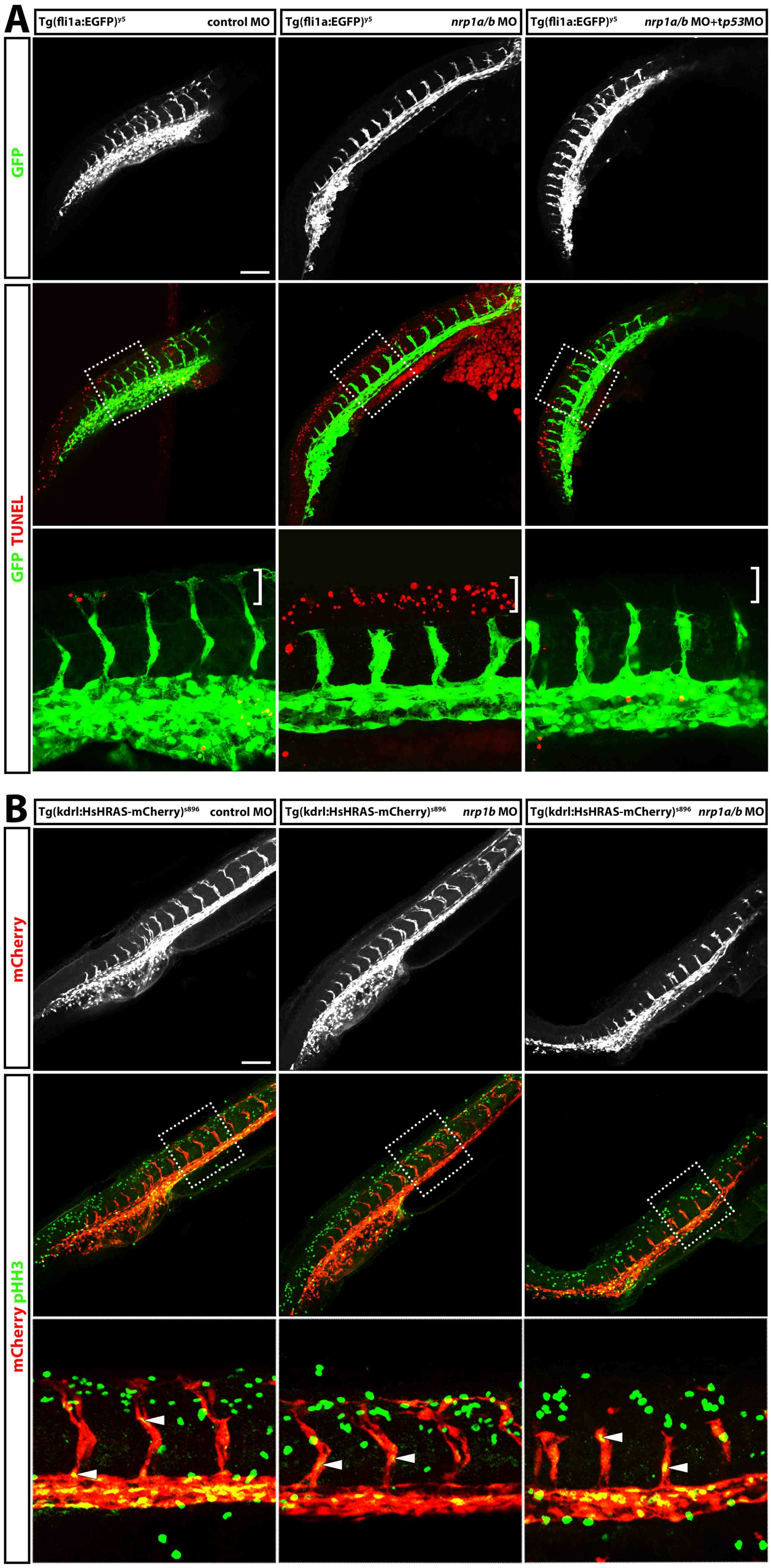
A
Tg(fli1a:EGFP)y5
control MO
Tg(fli1a:EGFP)y5
nrp1a/b MO
Tg(fli1a:EGFP)y5
nrp1a/b MO+tp53MO
GFP
GFP TUNEL
B
Tg(kdrl:HsHRAS-mCherry)s896
control MO
Tg(kdrl:HsHRAS-mCherry)s896
nrp1b MO
Tg(kdrl:HsHRAS-mCherry)s896
nrp1a/b MO
mCherry
mCherry pHH3

Figure S3

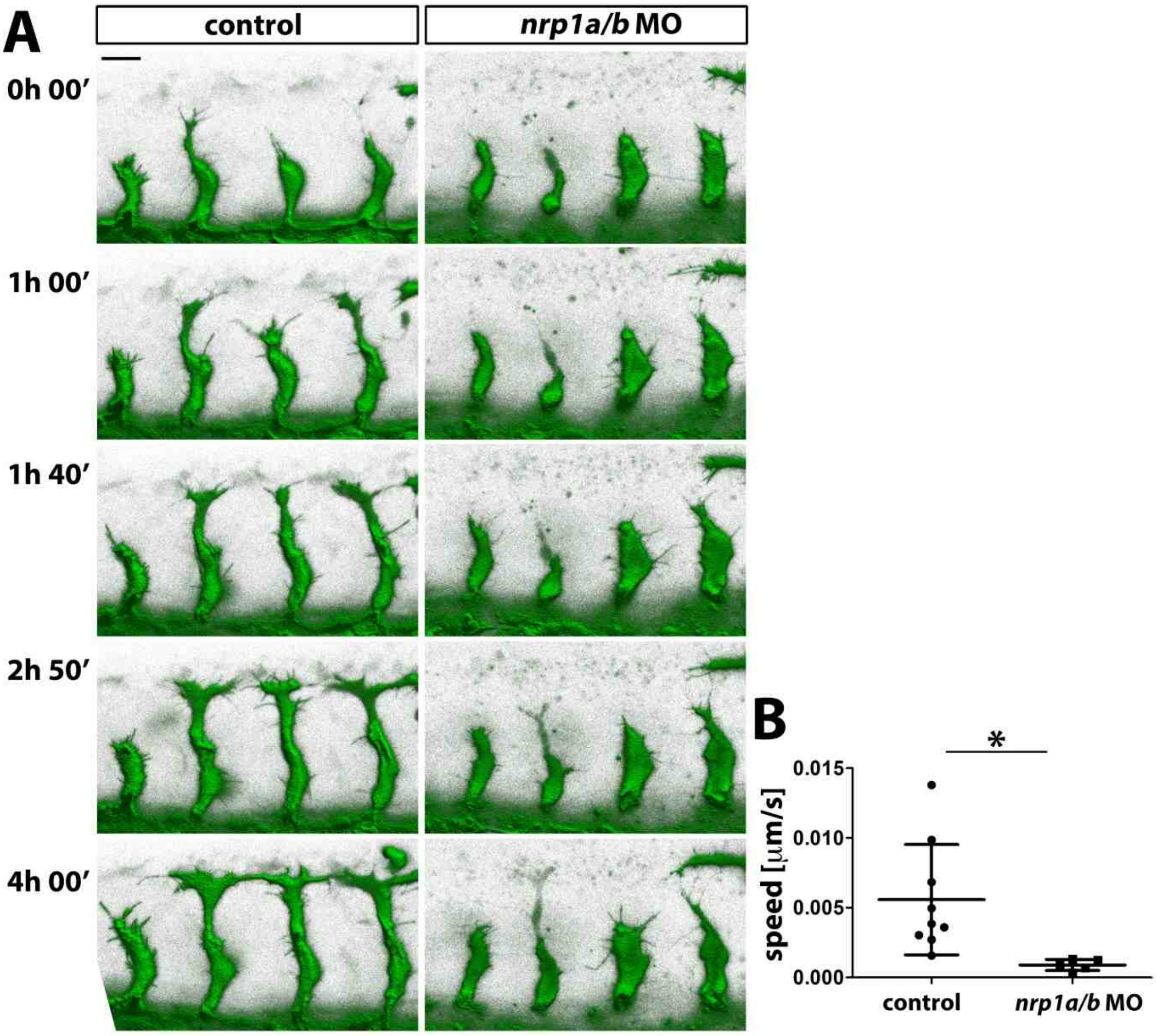
A
control
nrp1a/b MO
0h 00'
1h 00'
1h 40'
2h 50'
4h 00'
B
*
speed [μm/s]
0.015
0.010
0.005
0.000
control
nrp1a/b MO
