# Supplementary figures and images for: NRP1 Regulates CDC42 Activation to Promote Filopodia Formation in Endothelial Tip Cells
Source: Cell Rep. 2015 Jun 4;11(10):1577–90. doi: 10.1016/j.celrep.2015.05.018 (PMC4528263; doi:10.1016/j.celrep.2015.05.018)

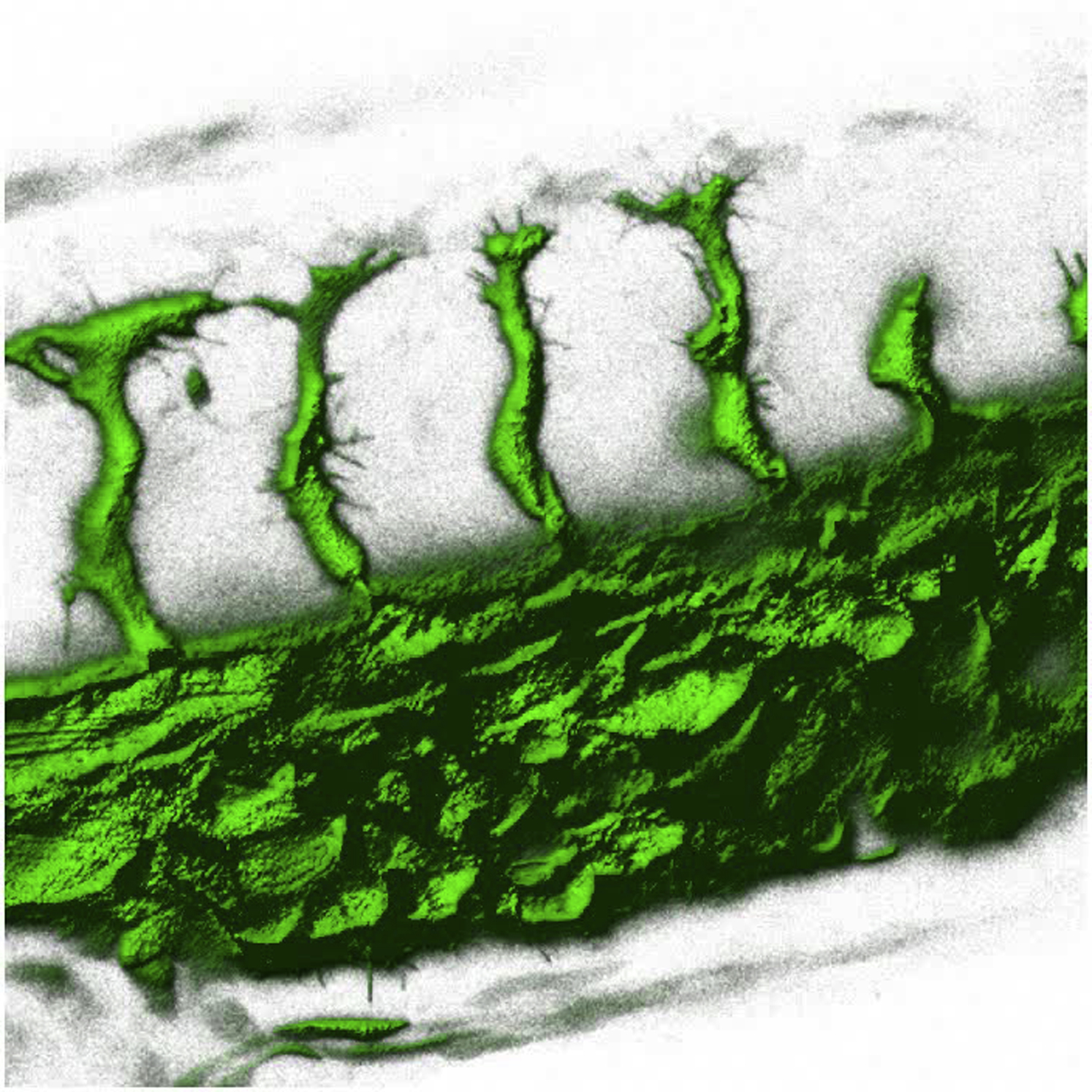

Supplement: Movie S1. Migration Speed of ISV Sprouts in Control Embryos, Related to Figures 5 and S3 [file mmc2.jpg]

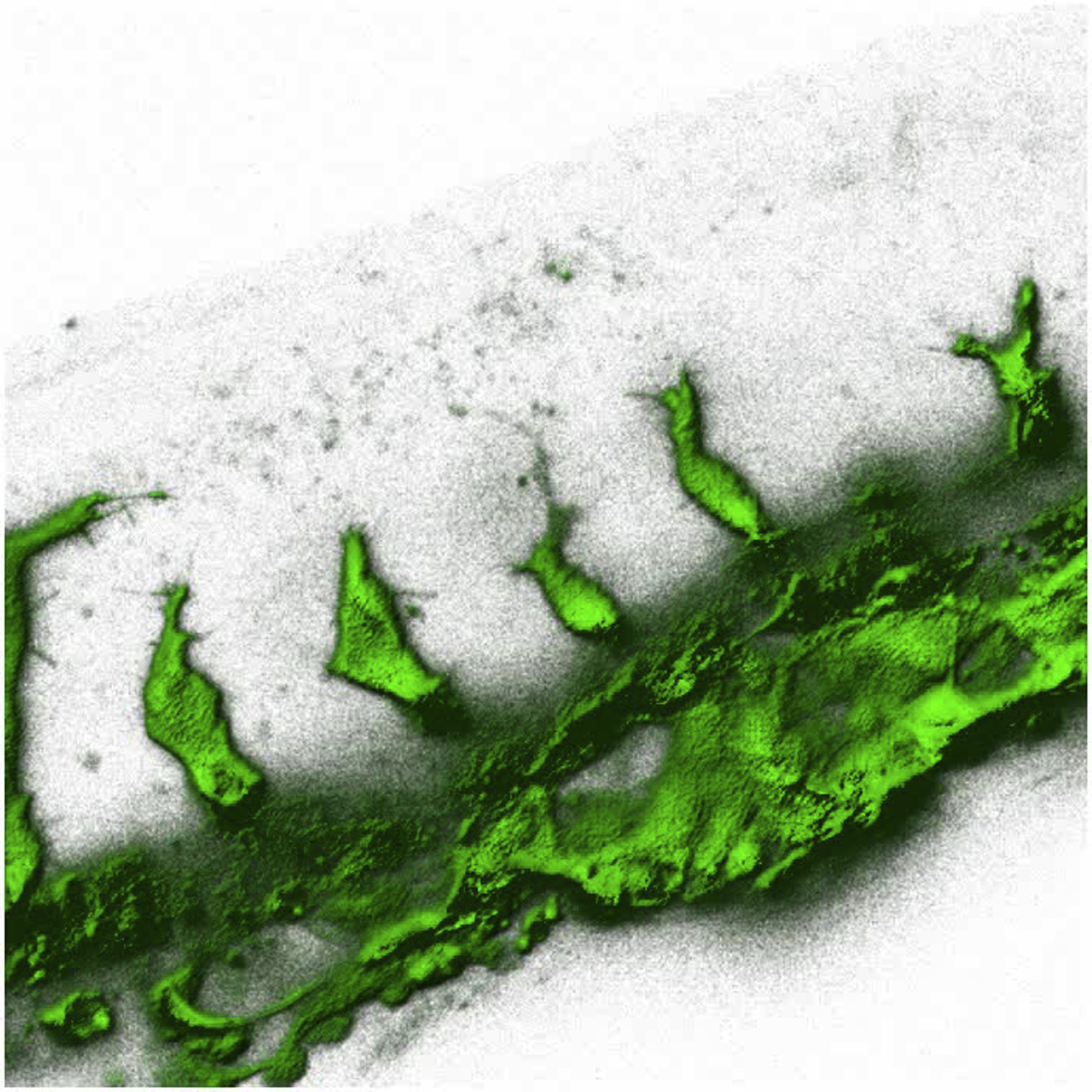

Supplement: Movie S2. Migration Speed of ISV Sprouts after Nrp1 Knockdown, Related to Figures 5 and S3 [file mmc3.jpg]
