# Supplementary material for: NRP1 Regulates CDC42 Activation to Promote Filopodia Formation in Endothelial Tip Cells
Source: Cell Rep. 2015 Jun 4;11(10):1577–90. doi: 10.1016/j.celrep.2015.05.018 (PMC4528263; doi:10.1016/j.celrep.2015.05.018)
Supplement: Document S2. Article plus Supplemental Information [file mmc4.pdf]

Article

Cell Reports

NRP1 Regulates CDC42 Activation to Promote Filopodia Formation in Endothelial Tip Cells

Graphical Abstract

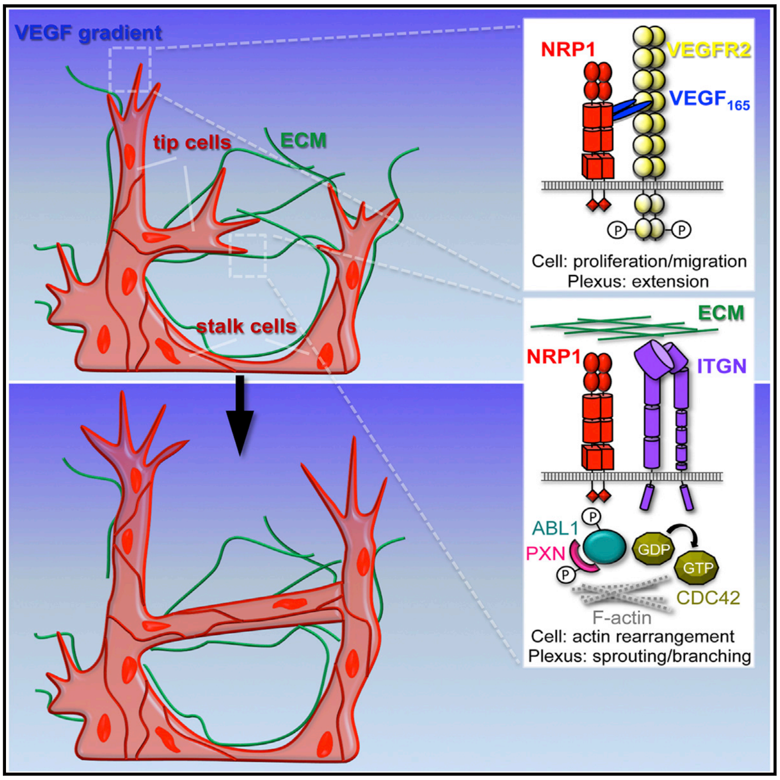

Highlights

- NRP1 enables morphological, but not genetic, specialization of endothelial tip cells
- NRP1 is essential for matrix-induced CDC42 activation in endothelial cells
- NRP1 promotes filopodia extension and actin remodeling via CDC42
- Endothelial NRP1 loss and CDC42 inhibition similarly impair angiogenesis

Authors

Alessandro Fantin,
Anastasia Lampropoulou, Gaia Gestri, ...,
Valentina Senatore, Ian Zachary,
Christiana Ruhrberg

Correspondence

c.ruhrberg@ucl.ac.uk

In Brief

During angiogenesis, new blood vessel sprouts are led by filopodia-studded tip cells to sense environmental signals and enable directional migration. NRP1 is a tip cell protein that senses angiogenic cues. Fantin et al. now show that NRP1 promotes tip cell function by enabling CDC42 activation for filopodia formation and actin remodeling.

Fantin et al., 2015, Cell Reports *11*, 1577–1590
June 16, 2015 ©2015 The Authors
http://dx.doi.org/10.1016/j.celrep.2015.05.018

Cell Reports
Article

OPEN ACCESS
CellPress

NRP1 Regulates CDC42 Activation to Promote Filopodia Formation in Endothelial Tip Cells

Alessandro Fantin,[1,4] Anastasia Lampropoulou,[1,4] Gaia Gestri,[2] Claudio Raimondi,[1] Valentina Senatore,[1] Ian Zachary,[3] and Christiana Ruhrberg[1,*]
[1]UCL Institute of Ophthalmology, University College London, 11-43 Bath Street, London EC1V 9EL, UK
[2]UCL Department of Cell and Developmental Biology, University College London, Gower Street, London WC1E 6BT, UK
[3]UCL Division of Medicine, University College London, 5 University Street, London WC1E 6JJ, UK
[4]Co-first author
*Correspondence: c.ruhrberg@ucl.ac.uk
http://dx.doi.org/10.1016/j.celrep.2015.05.018
This is an open access article under the CC BY license (http://creativecommons.org/licenses/by/4.0/).

SUMMARY

Sprouting blood vessels are led by filopodia-studded endothelial tip cells that respond to angiogenic signals. Mosaic lineage tracing previously revealed that NRP1 is essential for tip cell function, although its mechanistic role in tip cells remains poorly defined. Here, we show that NRP1 is dispensable for genetic tip cell identity. Instead, we find that NRP1 is essential to form the filopodial bursts that distinguish tip cells morphologically from neighboring stalk cells, because it enables the extracellular matrix (ECM)-induced activation of CDC42, a key regulator of filopodia formation. Accordingly, NRP1 knockdown and pharmacological CDC42 inhibition similarly impaired filopodia formation in vitro and in developing zebrafish in vivo. During mouse retinal angiogenesis, CDC42 inhibition impaired tip cell and vascular network formation, causing defects that resembled those due to loss of ECM-induced, but not VEGF-induced, NRP1 signaling. We conclude that NRP1 enables ECM-induced filopodia formation for tip cell function during sprouting angiogenesis.

INTRODUCTION

Developing organs, ischemic tissues, and growing tumors produce the vascular endothelial growth factor VEGF-A to signal to its receptors on the endothelial cells (ECs) that line all blood vessels, and the resulting angiogenic expansion of local vasculature ensures the delivery of oxygen and nutrients to sustain fundamental metabolic processes (Potente et al., 2011). VEGF-A signaling induces both the expansion of the EC pool by proliferation and the migration of ECs away from the existing plexus, whereby newly formed vessel sprouts are led by specialized tip cells that subsequently fuse to enable the formation of new vascular circuits (Fantin et al., 2010; Gerhardt et al., 2003; Ruhrberg et al., 2002). The highly polarized endothelial tip cells can be distinguished from neighboring stalk cells by clusters of numerous long filopodia that are thought to detect microenvironmental cues for directional migration (De Smet et al., 2009). Filopodia are highly dynamic cellular protrusions that contain parallel bundles of filamentous actin (F-actin) and can extend from lamellipodia (Mattila and Lappalainen, 2008). In addition to sensing growth factors, filopodia can adhere to the extracellular matrix (ECM) and form focal contacts that link the cytoskeleton to the ECM to promote forward movement.

The main regulators of filopodia formation are members of the RHO-GTPase family, in particular CDC42, which is activated by VEGF-A signaling in cultured ECs (Lamalice et al., 2004). Agreeing with a role for CDC42 in endothelial actin dynamics, both general and endothelial-specific CDC42 deletions disrupt blood vessel formation at the stage of vasculogenesis during mouse development (Chen et al., 2000; Jin et al., 2013). However, the resulting early embryonic lethality of these mutants has precluded investigations into the role of CDC42 in filopodia formation, tip cell function, and sprouting angiogenesis in vivo. Moreover, it is not known if VEGF-A and/or ECM cues are important for CDC42 regulation during vessel sprouting.

Neuropilin 1 (NRP1) is a non-tyrosine kinase transmembrane protein that regulates vascular development through dual roles in endothelial VEGF-A and ECM signaling (Fantin et al., 2014; Kawasaki et al., 1999; Lanahan et al., 2013; Raimondi et al., 2014). Using the mouse embryo hindbrain as a model to study physiological angiogenesis, we recently demonstrated a cell-autonomous requirement for NRP1 in endothelial tip cells during angiogenic sprouting (Fantin et al., 2013a). However, the specific cellular and molecular mechanisms that depend on NRP1 in tip cells have remained undefined. The prevailing model suggests that NRP1 acts as a VEGFR2 co-receptor downstream of VEGF-A signaling, which is chemotactic and induces the expression of essential tip cell genes. Supporting this idea, NRP1 can interact with VEGFR2 in ECs in vitro to potentiate VEGF-A signaling (e.g., Soker et al., 2002), and tip cell identity is promoted by VEGF-A signaling through VEGFR2 (Jakobsson et al., 2010). Alternatively, NRP1 may modulate signal transduction pathways that directly regulate tip cell behavior, such as cytoskeletal remodeling and filopodia extension. In agreement, NRP1 regulates filopodia orientation in hindbrain blood vessels (Gerhardt et al., 2004) and enables actin remodeling for EC migration via ABL kinases (Raimondi et al., 2014). However, it is not known how NRP1 might control filopodia formation and tip cell behavior.

OPEN
ACCESS
CellPress

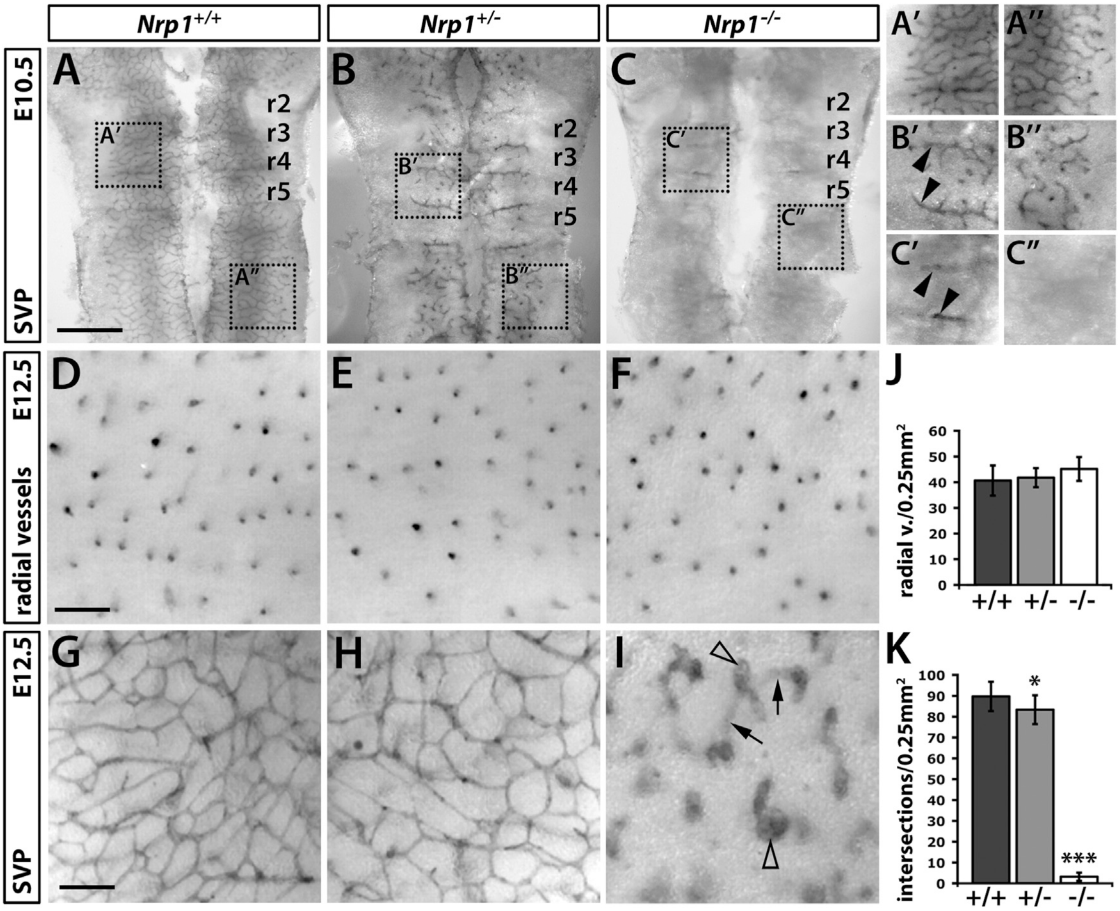

Figure 1. NRP1 Promotes Vessel Ingression Into and Growth Within the Neural Parenchyma

(A–C) PECAM immunohistochemistry of E10.5 mouse hindbrains of the indicated genotypes, flat-mounted with the ventricular side facing up; the position of individual rhombomeres (r) is indicated. Scale bar, 500 μm. (A′–C″) Higher magnification of the boxed areas in (AC); arrowheads indicate vessels in rhombomere boundaries.

(D–I) PECAM immunohistochemistry of E12.5 hindbrains of the indicated genotypes, flat-mounted to visualize radial (D–F) or SVP (G–I) vessels. Clear arrowheads indicate vascular tufts and arrows vascular bridges between radial vessels in deep brain layers (below the focal plane). Scale bar, 100 μm.

(J and K) Quantitation of radial vessel (J) or SVP branchpoint (K) number at E12.5, shown as mean ± SD, n ≥ 8 hindbrains each; asterisks indicate *$p < 0.05$ and ***$p < 0.001$.

Here, we have combined the analysis of vascular development in the mouse hindbrain with functional studies in primary human ECs, zebrafish embryos, and mouse retina to demonstrate that NRP1 is dispensable for the genetic specification of tip cells but essential for CDC42 activation. Unexpectedly, NRP1 enabled CDC42-dependent actin remodeling and filopodia formation in endothelial tip cells independently of VEGF-A stimulation, and loss of CDC42 activation did not phenocopy the vascular defects of mice with impaired VEGF-A signaling through NRP1. Instead, NRP1-mediated CDC42 activation was induced by stimulation with ECM, and loss of this pathway caused defective vessel sprouting and branching similar to loss of the ECM-induced, NRP1-dependent activation of ABL kinases. In addition to demonstrating a physiological role for ECM-induced CDC42 activation in sprouting angiogenesis, our work has therefore identified a mechanism that ensures the integration of growth factor signals with ECM cues via NRP1 to enable tip cell function in sprouting angiogenesis.

RESULTS

NRP1 Promotes Vessel Ingression into and Branching within the Neural Parenchyma

Using the mouse embryo hindbrain as a model for angiogenesis (Fantin et al., 2013b), we have recently shown that NRP1 confers a selective advantage to ECs competing for the tip cell position in growing vessel sprouts (Fantin et al., 2013a). To define specific roles for NRP1 in endothelial tip cells during angiogenesis, we first determined the emergence and nature of vascular defects in *Nrp1*-deficient mouse embryo hindbrains in greater detail than previously done. Immunohistochemistry for the vascular endothelial marker platelet endothelial cell adhesion molecule (PECAM) showed that vessel ingression was mildly delayed in hindbrains carrying one, and severely delayed in hindbrains carrying two, *Nrp1* null alleles (Figures 1A–1C). Thus, the wild-type subventricular vascular plexus (SVP) appeared well developed

at embryonic day 10.5 (E10.5) (Figures 1A′ and 1A″) but was less dense in heterozygous mutants (Figures 1B′ and 1B″) and had not formed in homozygous mutants at this stage (Figures 1C′ and 1C″). Instead, vessel growth in homozygous mutants was largely restricted to the boundary regions between neighboring hindbrain segments. The preferential vascularization of regions corresponding to rhombomere boundaries may reflect their high content of growth-factor-binding proteoglycans (Heyman et al., 1993, 1995) and agrees with findings in zebrafish (Ulrich et al., 2011). Importantly, heterozygous and homozygous mutants, similar to wild-type littermates, had an average of 35 somites at E10.5, suggesting that neither were affected by a general developmental delay at this stage. The comparison of wild-type, heterozygous, and homozygous mutant hindbrains therefore demonstrates a dose-dependent role for NRP1 in promoting vessel ingression into neural parenchyma.

Despite the defect in subventricular zone vascularization at E10.5, PECAM-labeled hindbrains of all genotypes had a similar number of radial vessels at E12.5 (Figures 1D–1F and 1J). Vessel ingression into the brain is therefore delayed in the absence of NRP1, but not irreversibly compromised. At E12.5, heterozygous mutants had formed an extensive SVP with a small but statistically significant reduction in branchpoints (Figures 1G, 1H, and 1K). Immunolabeling with a previously validated antibody for NRP1 (Fantin et al., 2010) showed reduced NRP1 levels in heterozygous compared to wild-type brains, especially in vessel sprouts (Figure S1, related to Figure 1), which correlated with reduced mRNA levels (see below). In contrast to heterozygous mutants, homozygous mutants lacked the SVP and instead formed large vascular tufts in the subventricular zone (Figure 1I, clear arrowheads), as previously reported (Fantin et al., 2013a; Gerhardt et al., 2004). Even though vessels had not branched in the appropriate plane for SVP formation, some radial vessels branched in deeper brain layers, where they formed vascular bridges that appeared out of focus in flat-mount preparations (Figure 1I, arrows). Altogether, this detailed characterization of hindbrain defects shows that NRP1 dose-dependently promotes vessel ingression into the brain as well as subventricular zone vascularization, two processes that specifically require angiogenic sprout extension.

Tip Cell Filopodia Defects in Homozygous and Heterozygous Nrp1 Null Mutants

Because NRP1 is particularly abundant on tip cell filopodia in hindbrain ECs (Fantin et al., 2013a), we next investigated if NRP1 deficiency affects either the formation of tip cells or their filopodia by using high-resolution confocal microscopy of isolectin B4 (IB4)-labeled E11.5 hindbrains. The wild-type hindbrain vasculature at this stage consisted of stalk cells with scattered filopodia and tip cells with elaborate filopodial bursts (Figures 2A and 2A′). Heterozygous mutants also formed many tip cells, but their filopodial bursts appeared less prominent than those of wild-type littermates (compare Figures 2A and 2A′ with Figures 1B and 1B′). Tip cells in homozygous mutants were difficult to identify by visual inspection, as only few filopodia extended from vessel termini, where tip cells normally reside (Figures 2C and 2C′). Quantification established that heterozygous mutants contained a similar density of morphologically identifiable tip cells compared to wild-types, while homozygous mutants had only a few obvious tip cells (Figure 2D).

Next, we adapted Imaris Filament Tracer software to automatically select IB4+ filopodia extending from IB4+ vessels in the different genotypes for quantitative analysis (Figures 2E, 2E′, 2F, and 2F′). This method demonstrated that the endothelial filopodia number was mildly but significantly reduced in heterozygous mutants and severely reduced in homozygous mutants relative to wild-type (Figure 2G). Filopodia tracing also allowed us to measure the dimensions of individual filopodia (Figures 2H–2I). Whereas the average filopodia length was not altered in *Nrp1* mutants (Figure 2H), the relative filopodial thickness was significantly reduced in heterozygous and more so in homozygous mutants compared to wild-type (Figure 2I). The decrease in filopodia number and thickness correlated with the severity of the branching phenotype in both heterozygous and homozygous mutants at the subsequent stage, E12.5 (see Figure 1). NRP1 mutants are therefore already compromised at the stage of endothelial tip cell and filopodia formation, preceding the previously reported defect of NRP1 mutants in filopodia guidance (Gerhardt et al., 2004).

NRP1 Is Dispensable for the Molecular Specification of Tip Cells

To investigate whether defects in endothelial tip cell and filopodia formation were a consequence of reduced tip cell molecular specification, we performed qPCR of genes known to be upregulated in tip cells in E11.5 hindbrains. For this analysis, we first validated the *Nrp1* knockdown in mutants and established the expression level of *Pecam* relative to the housekeeping gene *Actb* to provide a measure of EC quantity. Consistent with results obtained by immunolabeling (Figure S1, related to Figure 1), *Nrp1* mRNA levels were significantly decreased in heterozygous mutants (Figure 3A; mean fold change relative to control ± SD, $n \geq 3$: *Nrp1*$^{+/+}$ 1 ± 0.11, *Nrp1*$^{+/-}$ 0.63 ± 0.15; $p < 0.05$). As expected, *Nrp1* expression was not detectable in homozygotes due to the null mutation (Figure 3A). Correlating with decreased *Nrp1* levels and the NRP1 dose-dependent delay in brain vascularization (see above), *Pecam* levels were significantly decreased in both types of mutants, with a milder defect in heterozygotes (Figure 3B; mean fold change relative to control ± SD, $n \geq 3$: *Nrp1*$^{+/+}$ 1 ± 0.05, *Nrp1*$^{+/-}$ 0.84 ± 0.07, *Nrp1*$^{-/-}$ 0.68 ± 0.05; $p < 0.01$ for *Nrp1*$^{+/-}$ and *Nrp1*$^{-/-}$ relative to *Nrp1*$^{+/+}$). Similar results were obtained for another endothelial gene, VE-cadherin (*Cdh5;* mean fold change relative to control ± SD, $n \geq 3$: *Nrp1*$^{+/+}$ 1 ± 0.11, *Nrp1*$^{+/-}$ 0.78 ± 0.16, *Nrp1*$^{-/-}$ 0.69 ± 0.18; $p < 0.05$ for *Nrp1*$^{-/-}$ relative to *Nrp1*$^{+/+}$).

Next, we determined the expression level of several genes known to be upregulated in tip cells, including *Apln* and *Ang2* (del Toro et al., 2010), *Vegfr2* (Gerhardt et al., 2003), and *Dll4* (Hellström et al., 2007; Lobov et al., 2007). However, none of the tip cell markers examined showed decreased expression in *Nrp1* mutants. Thus, expression levels were similar across all three genotypes when normalized to *Actb*, with the exception of *Ang2*, which, rather than being decreased, was actually significantly increased in *Nrp1*$^{-/-}$ mice (mean fold change relative to control ± SD: *Apln*: *Nrp1*$^{+/+}$ 1 ± 0.08, *Nrp1*$^{+/-}$ 0.9 ± 0.04, *Nrp1*$^{-/-}$ 1.13 ± 0.19, $p > 0.05$; *Vegfr2*: *Nrp1*$^{+/+}$ 1 ± 0.13,

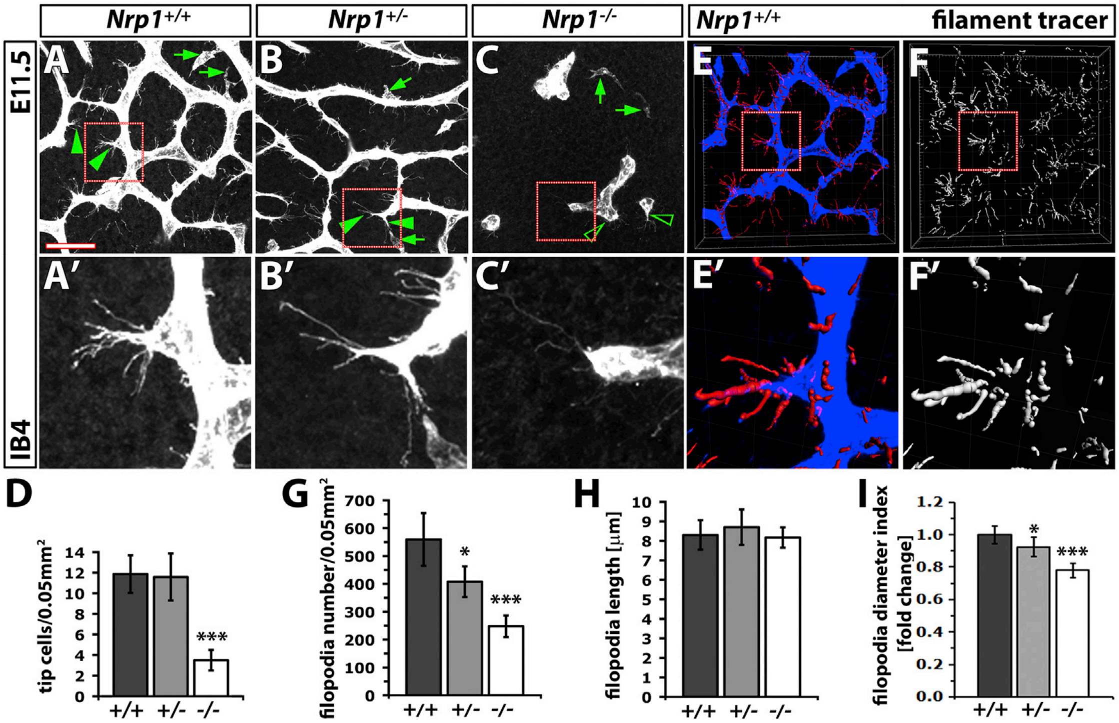

Figure 2. NRP1 Regulates Tip Cell Morphology in a Dose-Dependent Manner

(A–C) IB4-labeled E11.5 littermate hindbrains of the indicated genotypes; green arrowheads and arrows indicate examples of tip cells and macrophages, respectively. Scale bar, 50 μm. (A′–C′) Higher-magnification images of the boxed areas in (AC).

(D) Quantitation of tip cell number in wild-type and *Nrp1* null hindbrains, shown as mean ± SD; $Nrp1^{+/+}$ and $Nrp1^{+/-}$ n = 7 hindbrains each, $Nrp1^{-/-}$ n = 3 hindbrains; asterisks indicate ***p < 0.001.

(E and F) Confocal z stack of a wild-type hindbrain, processed with Imaris filament tracer for filopodia analysis. In (E), the vessel plexus and filopodia are shown in blue and red, respectively. In (F), the filopodia tracks are shown in white. (E′ and F′) Higher-magnification images of the boxed areas in (E) and (F).

(G–I) Quantitation of filopodia number, length, and thickness index with Imaris filament tracer, shown as mean ± SD, n ≥ 3 hindbrains; asterisks indicate *p < 0.05 and ***p < 0.001.

$Nrp1^{+/-}$ 0.9 ± 0.07, $Nrp1^{-/-}$ 0.98 ± 0.24, p > 0.05; *Dll4*: $Nrp1^{+/+}$ 1 ± 0.11, $Nrp1^{+/-}$ 1.01 ± 0.13, $Nrp1^{-/-}$ 1.04 ± 0.1, p > 0.05; *Ang2*: $Nrp1^{+/+}$ 1 ± 0.11, $Nrp1^{+/-}$ 1.01 ± 0.14, $Nrp1^{-/-}$ 1.34 ± 0.19, p < 0.05; all p values refer to $Nrp1^{-/-}$ relative to $Nrp1^{+/+}$). When normalized to *Pecam* as a measure of overall vascular volume, the mRNA expression of all tip cell markers examined, including that of the notch ligand DLL4, was significantly increased in homozygous mutants (Figures 3C–3F). Agreeing with increased *Dll4* expression, the expression of the notch targets *Hes1* and *Hey1* (Phng et al., 2009) was also slightly increased in *Nrp1* mutants (Figures 3G and 3H).

These findings suggest that NRP1 is dispensable for the genetic specification of tip cells. Instead, tip cell marker expression was upregulated in the absence of NRP1, consistent with a more immature vessel plexus and, possibly, gene-regulatory compensation for non-productive vessel sprouting.

NRP1 Is Required for ECM-Induced CDC42 Activation in Primary Human ECs

The essential role for NRP1 in tip cell filopodia formation, but not tip cell specification, raised the possibility that NRP1 promotes the tip cell phenotype via CDC42, a small RHO-GTPase that cycles between a GTP-bound active and a GDP-bound inactive state to regulate actin cytoskeleton reorganization, filopodia extension, and directional migration in other cell types (Heasman and Ridley, 2008). To investigate if NRP1 promotes filopodia extension by regulating CDC42 activation, we used primary ECs as an in vitro model that is amenable to both cell biological and biochemical studies. For these experiments, we chose human dermal microvascular endothelial cells (HDMECs), because dermal vasculature naturally undergoes extensive angiogenesis during wound healing, and because transfection of a previously validated small interfering RNA (siRNA) targeting NRP1 (si-NRP1) effectively knocks down NRP1 in these cells (Raimondi et al., 2014). To measure levels of GTP-bound (i.e., activated) CDC42, we used a pull-down assay with the p21-binding domain of the p21-activated protein kinase PAK1 (Benard et al., 1999). This experiment showed that stimulation with the ECM component fibronectin (FN) for 30 min efficiently activated CDC42 in HDMECs (Figure 4A). Moreover, ML141, a validated allosteric inhibitor with exquisite specificity for CDC42 over other small RHO-GTPases (Hong et al., 2013), effectively targeted this FN-dependent CDC42 activation, confirming specificity of the assay (Figure 4A).

We therefore transfected HDMECs with control siRNA (si-control) or NRP1 siRNA (si-NRP1), stimulated them with FN, and

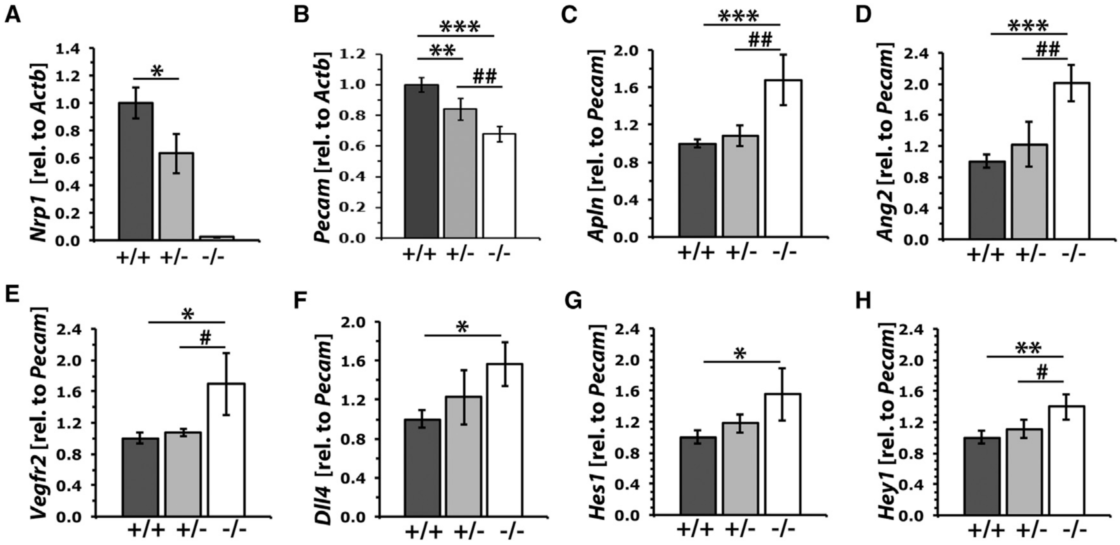

Figure 3. The Expression of Markers for Tip Cell Specification Is Not Impaired in *Nrp1* Mutants

(A and B) qPCR expression analysis of *Nrp1* (A) and *Pecam* (B), normalized to *Actb* in E11.5 hindbrains of the indicated genotypes (n = 4 each); expression levels are shown as mean ± SD in mutants relative to wild-type.

(C–H) qPCR expression analysis of the indicated tip cell markers and notch target genes in E11.5 hindbrains of the indicated genotypes (n = 4 each), normalized to *Pecam* to obtain an objective measure of tip cell marker expression relative to the amount of endothelium; expression levels are shown as mean ± SD in mutants relative to wild-type.

Asterisks indicate p values for homozygous or heterozygous mutants relative to wild-type (*p < 0.05, **p < 0.01,***p < 0.001), hash tags p values for homozygous relative to heterozygous mutants (#p < 0.05, ##p < 0.01).

compared levels of total and activated CDC42 with this method. While NRP1 knockdown did not affect the overall level of CDC42 (Figure 4B), it efficiently inhibited FN-induced CDC42 activation (Figure 4C). Reduced CDC42 activation was also observed in cells lacking NRP1 when VEGF-A was provided as an additional stimulus (Figure 4D). In a parallel approach, we measured levels of GTP-bound, activated CDC42 by performing pull-down assays with the CDC42-binding domain of Wiskott-Aldrich syndrome protein (WASP) fused to glutathione S-transferase (GST) (GST-WASP) (Kolluri et al., 1996). These experiments confirmed that FN stimulation increases CDC42 activation in HDMECs (Figure 4E, first two lanes) and that NRP1 was required for normal CDC42 activation after FN stimulation (Figure 4E, middle lanes), as shown with the GST-PAK1 assay (Figure 4C). As expected, ML141 inhibited CDC42 activation in this assay (Figure 4E, last two lanes), similar to the GST-PAK1 assay (Figure 4A). The quantitative analysis of GTP-bound, activated CDC42 confirmed that ML141 treatment significantly decreased CDC42 activation, as expected (Figure 4F, left graph; mean fold change relative to control ± SD: control 1 ± 0.1 versus ML141 0.37 ± 0.17, $p < 0.05$). Moreover, there was a significant decrease in CDC42 activation in HDMECs transfected with si-NRP1 compared to si-control cells (Figure 4F, right graph; mean fold change relative to control ± SD: si-control 1 ± 0.11, si-NRP1 0.34 ± 0.24, $p < 0.05$; n = 3 independent experiments). In agreement with CDC42 being regulated by NRP1, CDC42 co-immunoprecipitated with NRP1 in HDMECs both before and during FN stimulation (Figure 4G).

Phalloidin staining for visualization of F-actin showed that many control cells after 2 hr on FN had assumed an elongated appearance with irregular edges and numerous stress fibers, typical of motile cells (Figure 4H). In contrast, NRP1 knockdown caused many cells to adopt a rounded morphology with few stress fibers (Figure 4I), as previously shown (Raimondi et al., 2014). A similar phenotype was induced by ML141-mediated CDC42 inhibition (Figures 4J and 4K). Higher-magnification images showed that the altered morphology of NRP1-deficient and ML141-treated cells correlated with reduced cell protrusive activity compared to control cells (Figures 4H′, 4I’, and 4K′). Quantitative analysis confirmed that both NRP1 knockdown and CDC42 inhibition significantly reduced the number of actin-positive, filopodia-like microspikes extending from the cell periphery in HDMECs plated on FN for 1 hr (Figures 4L and 4M).

Additional VEGF-A stimulation further increased the number of microspikes in cells plated on FN compared to cells plated on FN without VEGF-A stimulation (Figures 4L and 4M, compare dark gray columns). VEGF-A addition also increased the number of microspikes in NRP1-depleted ECs on FN (Figure 4L, compare light gray columns). Yet, the relative reduction in the number of microspikes between si-control and si-NRP1 cells was similar in both FN-only and FN+VEGF conditions (Figure 4L, red arrows). Moreover, the relative reduction in microspike number was similar in ML141-treated compared to NRP1 knockdown HDMECs on FN (compare Figure 4L with Figure 4M). Surprisingly, however, ML141 treatment was less effective than NRP1 knockdown in reducing the microspike number of cells plated on FN and stimulated with VEGF-A compared to cells on FN without VEGF-A stimulation (Figure 4M). This observation raised the possibility that VEGF-A can stimulate microspike formation in both CDC42-dependent and CDC42-independent

OPEN ACCESS
CellPress

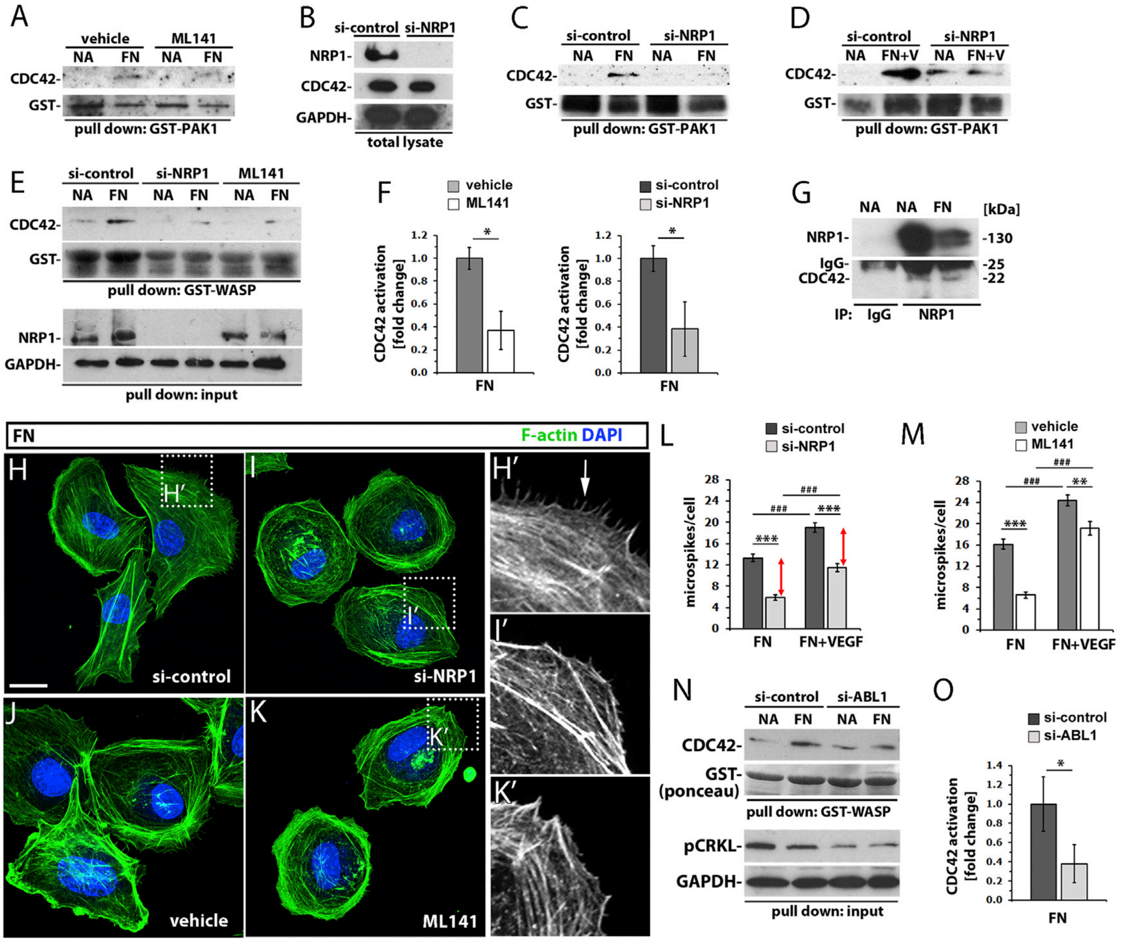

Figure 4. NRP1 Is Required for Normal CDC42 Activation, and CDC42 or NRP1 Knockdown Similarly Impairs Actin Remodeling in Primary Human ECs

(A–E) NRP1 enables ECM-induced CDC42 activation. HDMECs were serum-starved and treated with vehicle or ML141 for 30 min (A and E) or transfected with si-control or si-NRP1 and serum starved (B–E); protein lysates of non-adherent (NA) cells or adherent cells after 30 min on FN were incubated with PAK1-GST (A, C, and D) or WASP-GST (E, top) beads and immunoblotted or used directly for immunoblotting (B and E, bottom). In (D), HDMECs were additionally stimulated for 15 min with 5 ng/ml VEGF165 (FN+V).

(F) Impaired CDC42 activation after ML141 treatment or NRP1 knockdown in ECM-stimulated ECs. Activated CDC42 was normalized to GST input and expressed as mean fold change relative to control ± SD; n = 3; asterisks indicate *p < 0.05.

(G) Complex formation of endogenous NRP1 and CDC42 in ECs. Lysates from NA and FN-adherent HDMECs were immunoprecipitated with control immunoglobulin G (IgG) or NRP1 antibody, followed by immunoblotting for NRP1 and CDC42. The 25-kDa IgG band is indicated.

(H–M) NRP1 and CDC42 are required for ECM-induced actin remodeling in ECs. After transfection with control versus NRP1 si-RNA or treatment with vehicle versus ML141, HDMECs were detached, plated on FN for 2 hr, and stained with phalloidin to label F-actin and DAPI to visualize cell nuclei. Scale bar, 20 μm. Higher-magnification images of the boxed areas in (H, I, and K) are shown in (H′), (I′), and (K′). Microspike quantitation after NRP1 knockdown (L) or CDC42 inhibition (M) and plating for 1 hr on FN, shown as mean microspike number per cell ± SEM; n ≥ 42 cells for each condition (asterisks indicate p values for control relative to si-NRP1- or ML141-treated cells: **p < 0.01, ***p < 0.001; hashtags indicate p values for FN without VEGF-A stimulation [FN] relative to cells on FN with additional VEGF-A stimulation [FN+VEGF], ###p < 0.001). Red arrows indicate the similar reduction in microspikes on FN compared to FN+VEGF.

(N and O) ABL1 enables CDC42 activation in ECM-stimulated ECs. After transfection with si-control or si-ABL1, lysates from NA and FN-adherent HDMECs were incubated with WASP-GST beads followed by immunoblotting (N, top) or used directly for immunoblotting (N, bottom). GST staining and GAPDH immunoblotting confirmed similar input of GST-beads and lysate. (O) Activated CDC42 was normalized to GST input and expressed as mean fold change relative to control ± SD; n ≥ 3; asterisks indicate *p < 0.05.

pathways, although this idea was not investigated further in the present study.

Taken together, our observations establish that NRP1 enables CDC42 activation and CDC42-dependent actin dynamics and filopodia extension in ECM-stimulated ECs, independently of VEGF-A signaling.

ABL1 Is Required for NRP1-Dependent CDC42 Activation

The phenotype of HDMECs after CDC42 inhibition or NRP1 knockdown resembled the cellular phenotype caused by ABL1 knockdown, including a rounded cell shape, increased cortical actin, reduced stress fibers, and impaired microspike

formation (Raimondi et al., 2014). Moreover, similar to CDC42 activation (Figures 4C–4F), ABL1 activation depends on NRP1 in FN-stimulated ECs (Raimondi et al., 2014). We therefore examined if ABL1 was upstream of CDC42 activation in FN-stimulated ECs. For this experiment, we transfected HDMECs with control siRNA (si-control) or siRNA targeting ABL1 (si-ABL1), as previously shown (Raimondi et al., 2014), stimulated the cells with FN, and then performed GST-WASP pull-down assays for activated CDC42. This experiment demonstrated that ABL1 loss attenuated FN-induced CDC42 activation (Figures 4N and 4O; mean fold change relative to control ± SD: si-control 1 ± 0.28 versus si-ABL1 0.38 ± 0.2, $p < 0.05$; n = 3 independent experiments). ABL1 knockdown was confirmed by reduced CRKL phosphorylation (pCRKL; Figure 4N), a known ABL1 kinase target (Lewis et al., 1996). The similar loss of CDC42 activation after ABL1 or NRP1 knockdown (compare Figure 4F with Figure 4O) is consistent with the idea that ABL1 is upstream of CDC42 activation in NRP1-mediated ECM signaling.

Nrp1 Deficiency and Cdc42 Inhibition Similarly Impair Vascular Sprout Extension in Zebrafish

To investigate whether NRP1 promotes the tip cell phenotype via CDC42 in vivo and to extend our findings on NRP1 to a model organism amenable to live imaging, we asked how Nrp1 knockdown and Cdc42 inhibition affected angiogenesis in the developing zebrafish trunk, where intersomitic vessels (ISVs) sprout from the dorsal aorta and then fuse into the dorsal longitudinal anastomosing vessels (DLAV) (Lawson and Weinstein, 2002). Zebrafish contain two *nrp1* homologs, *nrp1a* and *nrp1b*, and both are expressed in vascular endothelium and reported to regulate vascular development (Bovenkamp et al., 2004; Martyn and Schulte-Merker, 2004; Yu et al., 2004). However, the genetic targeting of *nrp1a* alone was recently suggested to not impair angiogenesis (Kok et al., 2015). This observation implies that Nrp1a acts redundantly with its homolog Nrp1b during ISV sprouting. We therefore sought to downregulate both Nrp1 homologs to disrupt Nrp1-mediated angiogenesis in zebrafish. For these experiments, we used a *nrp1a/b* morpholino (MO) that had originally been designed to target Nrp1 at a time when Nrp1a, but not Nrp1b, had been identified (Hillman et al., 2011; Lee et al., 2002) and is referred to as MO2-nrp1a in the zfin database; however, our bioinformatics analysis predicted that this MO might also target *nrp1b* (Figure 5A). To test this prediction, we performed immunoblotting with a previously validated antibody that recognizes an evolutionarily conserved 14-aa region in the cytoplasmic domain of human and mouse NRP1 (Fantin et al., 2011). Consistent with this region being conserved in the zebrafish Nrp1a and Nrp1b homologs (Figure 5B), this antibody identified a band of ~130 kDa corresponding to Nrp1 in protein lysates from control fish, but not fish treated with the *nrp1a/b* MO (Figure 5C).

Because Nrp1a and Nrp1b are similar in size, with 923-aa and 959-aa residues, respectively, we next separated protein lysates from fish embryos on a gradient gel followed by immunoblotting with the Nrp1 cytoplasmic tail antibody (Figure 5D). Using this method, we resolved two bands at ~130 kDa corresponding to the two Nrp1 homologs and confirmed that both proteins were knocked down after treatment with the *nrp1a/b* MO (Figure 5D). Moreover, the antibody detected only the band corresponding to Nrp1a in size after *nrp1b* knockdown (Figure 5D). Agreeing with the bioinformatics analysis, the *nrp1a/b* MO that is referred to as MO2-nrp1a in the zfin database therefore targets both Nrp1a and Nrp1b.

Having established that we could effectively downregulate both Nrp1 homologs, we imaged the trunk vasculature of control and *nrp1a/b* MO-treated *Tg(fli1a:EGFP)*^{y5} embryos expressing a fluorescent endothelial reporter (Lawson and Weinstein, 2002) at 32 hours post-fertilization (hpf) (Figure 5E). We observed that morphant fish lacking both Nrp1a and Nrp1b had a similar number of ISV sprouts overall compared to control fish (Figures 5E and 5F). However, the number of sprouts that had reached the dorsal trunk was severely reduced in morphants compared to controls and, consequently, sprouts had not fused to form the DLAV in embryos lacking Nrp1a and Nrp1b (Figures 5E, 5G, and 5H). In contrast, a control MO did not affect vascular development (Figures S2A and S2B, related to Figure 5). Moreover, the *nrp1b* MO alone, used at a dose effective to knockdown the protein (Figure 5D), did not cause severe vascular defects as in *nrp1a/b* MO-treated embryos (Figure S2B, related to Figure 5), in agreement with the idea that Nrp1a and Nrp1b have redundant functions during ISV sprouting.

Treatment with the *nrp1a/b* MO increased apoptosis in the neural tube compared to a control MO (Figure S2A, related to Figure 5), which may be due to a neuroprotective role for Nrp1 in neural progenitors that express Nrp1a (Bovenkamp et al., 2004; Lee et al., 2002; Martyn and Schulte-Merker, 2004; Yu et al., 2004) and/or unspecific MO toxicity. To exclude that neural tube apoptosis was indirectly responsible for the vascular defect of *nrp1a/b* morphants, we co-injected a *tp53* MO to suppress apoptosis (Paridaen et al., 2011). As the resulting suppression of apoptosis did not rescue the ISV defect phenotype of *nrp1a/b* morphants (Figure S2A, related to Figure 5), the ISV defect of *nrp1a/b* MO-injected fish cannot be explained by increased neural tube apoptosis. Instead, impaired ISV extension is likely caused by a cell-autonomous defect in endothelial cells deficient in NRP1, as observed for mouse vascular development (Fantin et al., 2013a).

Live imaging of *Tg(fli1a:EGFP)*^{y5} zebrafish embryos further allowed us to compare the process of ISV sprouting over time (Movies S1 and S2; Figure S3, related to the Movies S1 and S2 and Figure 5). We observed that the migration speed of ISV sprouts was significantly reduced in embryos injected with *nrp1a/b* MO compared to controls. This defect correlated with reduced filopodia formation and dynamics, especially at the sprout front, and, accordingly, the sprouts appeared to lack proper tip cells (Movies S1 and S2; Figure S3, related to Movies S1 and S2 and Figure 5). In contrast, staining for the mitotic marker phosphorylated histone H3 (pHH3) showed that ECs still proliferate in ISV sprouts of embryos lacking Nrp1a and Nrp1b, excluding that impaired sprout extension is caused by defective EC proliferation (Figure S2, related to Figure 5). The finding that Nrp1 is required for tip-cell-led endothelial motility during ISV sprout extension shows that NRP1's function as a positive

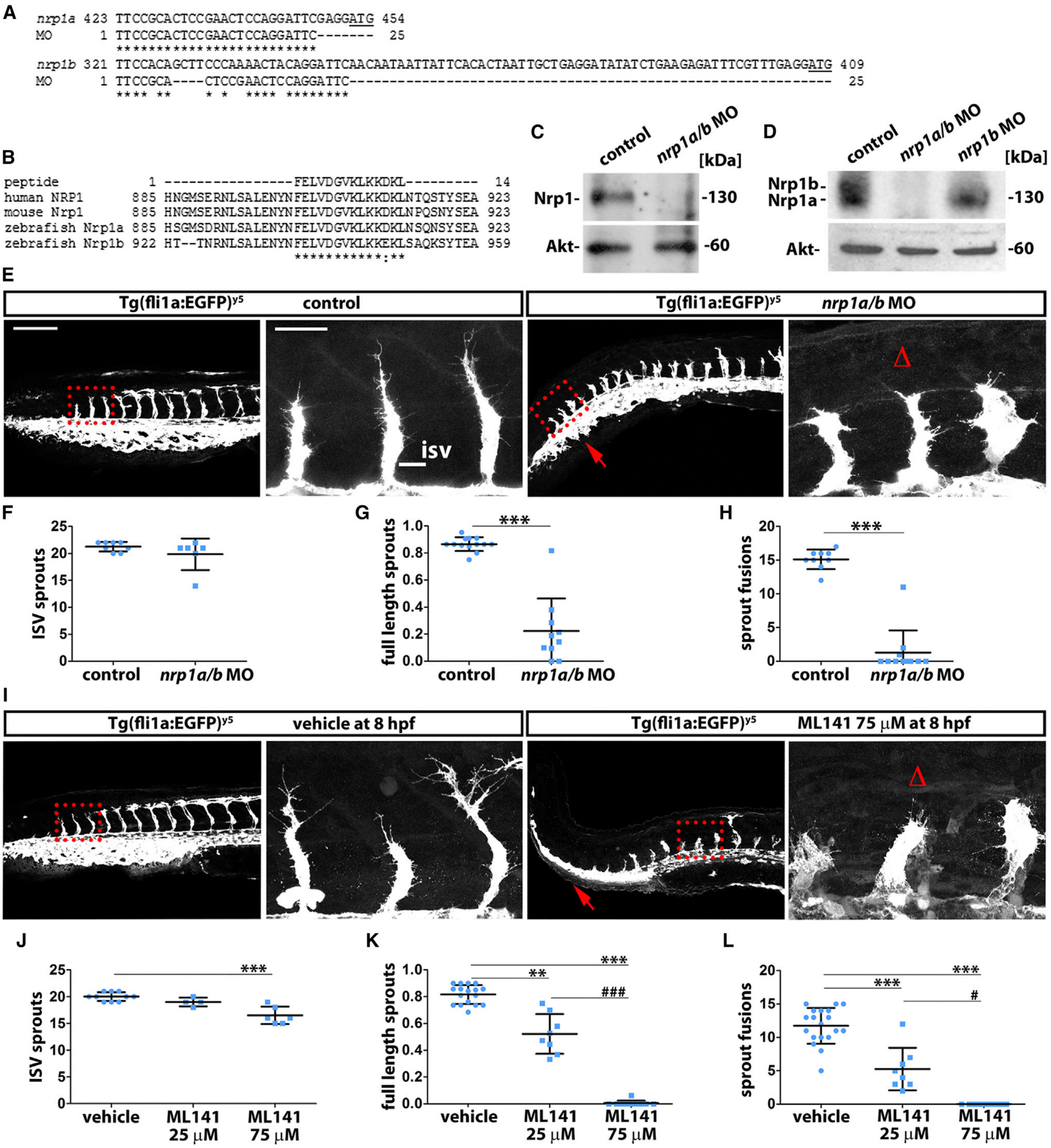

Figure 5. Vessel Sprouting Is Compromised by Nrp1 Knockdown or Cdc42 Inhibition in Zebrafish

(A) Alignment of the *nrp1a/b* MO nucleotide target sequence with the 5′ UTR immediately upstream of the start codon (underlined) in *nrp1a* and *nrp1b*; asterisks indicate identical nucleotides.

(B) Alignment of the peptide sequence recognized by anti-NRP1 antibody with the cytoplasmic tail of human and mouse NRP1 as well as zebrafish Nrp1a and Nrp1b; asterisks indicate identical amino acid residues, and the colon indicates a conservative substitution.

(C and D) Protein lysates from control and *nrp1a/b* and *nrp1b* MO-injected 32-hpf fish embryos were used for Nrp1 and Akt immunoblotting after electrophoresis using a 10% gel (C) or 4%–12% gradient gel (D).

(E–L) Confocal z stacks of trunks from 32 hpf *Tg(fli1a:EGFP)*^{y5} zebrafish and corresponding quantitation of the indicated vascular parameters for controls versus *nrp1a/b* MO (E–H) and vehicle versus ML141 treatment (I–L); scale bar, 200 μm. A kinked tail caused by Nrp1 knockdown is also seen after Cdc42 inhibition

(legend continued on next page)

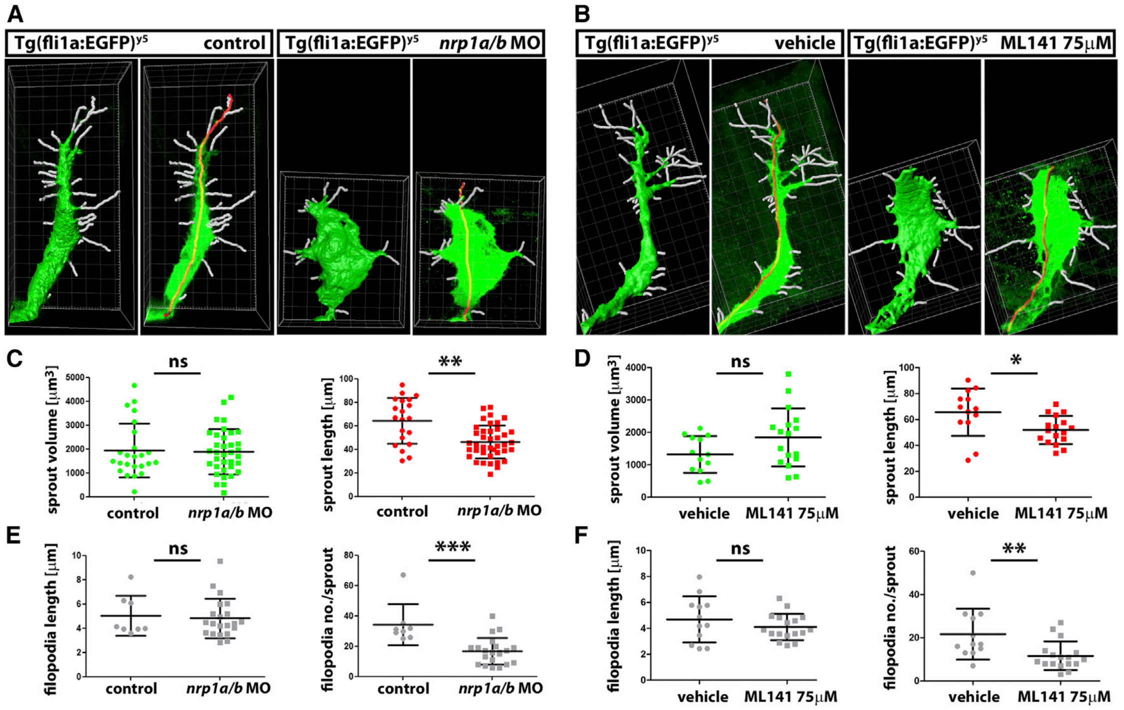

Figure 6. Cdc42 Inhibition Phenocopies Nrp1 Knockdown during Zebrafish Angiogenesis

(A and B) Imaris image analysis of ISV sprouts in *nrp1* morphant, ML141-treated and control embryos to determine sprout volume via surface rendering (left panel for each condition) and filament tracing to determine sprout length (red line in the right panel for each condition) and filopodia number as well as filopodia length (white lines). Grid ticks, 1 μm.

(C–F) Quantitative analysis of the Imaris images exemplified in (A and B) for sprout volume and sprout length (C and D), filopodia length and filopodia number (E and F); mean ± SD, n ≥ 8 sprouts for *nrp1* morphant relative to control treated fish (C and E) and ML141- versus vehicle-treated fish (D and F); asterisks indicate *p < 0.05, **p < 0.01, ***p < 0.001, and ns > 0.05.

modulator of vessel sprouting is conserved between fish and mice.

Next, we inhibited Cdc42 in *Tg(fli1a:EGFP)*^{y5} embryos by adding ML141 to the aquarium water at late gastrula stage (8 hpf) and imaged the trunk vasculature at 32 hpf (Figure 5I). Treatment with 25 μM ML141 did not reduce the number of ISV sprouts, but fewer vessels had reached the dorsal trunk than in vehicle-treated controls, and consequently, the DLAV had not formed at this stage (Figures 5J–5L). Treatment with 75 μM ML141 perturbed ISV formation more severely, with slightly fewer ISV sprouts and a catastrophic failure of sprouts to reach the dorsal trunk and form the DLAV (Figure 5I–5L), as observed in *nrp1* morphants (see Figures 5E–5H). Cdc42 inhibition therefore impairs angiogenesis in a dose-dependent fashion. The observation that targeting Nrp1 or Cdc42 affected sprout extension in the zebrafish trunk in a similar fashion supports the idea that both genes operate in a shared angiogenic pathway.

We used Imaris image analysis to quantify vascular sprout defects in *nrp1* knockdown and ML141-treated *Tg(fli1a:EGFP)*^{y5} zebrafish embryos relative to controls (Figure 6). Specifically, we surface rendered the three most caudal ISV sprouts in each fish embryo to determine their average volume (Figures 6A and 6B, left panel for each condition) and applied the filament tracer module to determine average sprout length (Figures 6A and 6B, right panel for each condition). We observed that the total ISV sprout volume was not affected by Nrp1 knockdown or Cdc42 inhibition but that sprout length was significantly reduced after either treatment compared to controls (Figures 6C and 6D). Filament tracer analysis of filopodia formation further showed that the average length of filopodia was similar in ISV sprouts of control and treated fish but that the filopodia number was significantly reduced after Nrp1 knockdown or Cdc42 inhibition (Figures 6E and 6F). The similar filopodia defects observed in hindbrain angiogenesis and zebrafish ISV sprouting after NRP1 loss suggest that the tip cell role of NRP1 is conserved across vertebrate species. Genome editing in fish to create double *Nrp1a/b* mutants may therefore be a useful next step for extended analyses of NRP1 function in angiogenesis.

(red arrows). Boxed areas are shown at higher magnification adjacent to each corresponding panel to illustrate delayed sprout extension; scale bar, 25 μm. Δ indicates impaired sprout invasion into the dorsal trunk. The number of all ISV sprouts (F and J), full-length ISV sprouts (G and K), or ISV sprouts that have fused laterally (H and L) is shown as mean ± SD (n ≥ 4 fish for each treatment condition). Asterisks indicate p values for *nrp1a/b* MO or ML141 relative to controls **p < 0.01, ***p < 0.001; hashtags indicate p values for 75 μM relative to 25 μM ML141 #p < 0.05, ###p < 0.001.

OPEN
ACCESS
CellPress

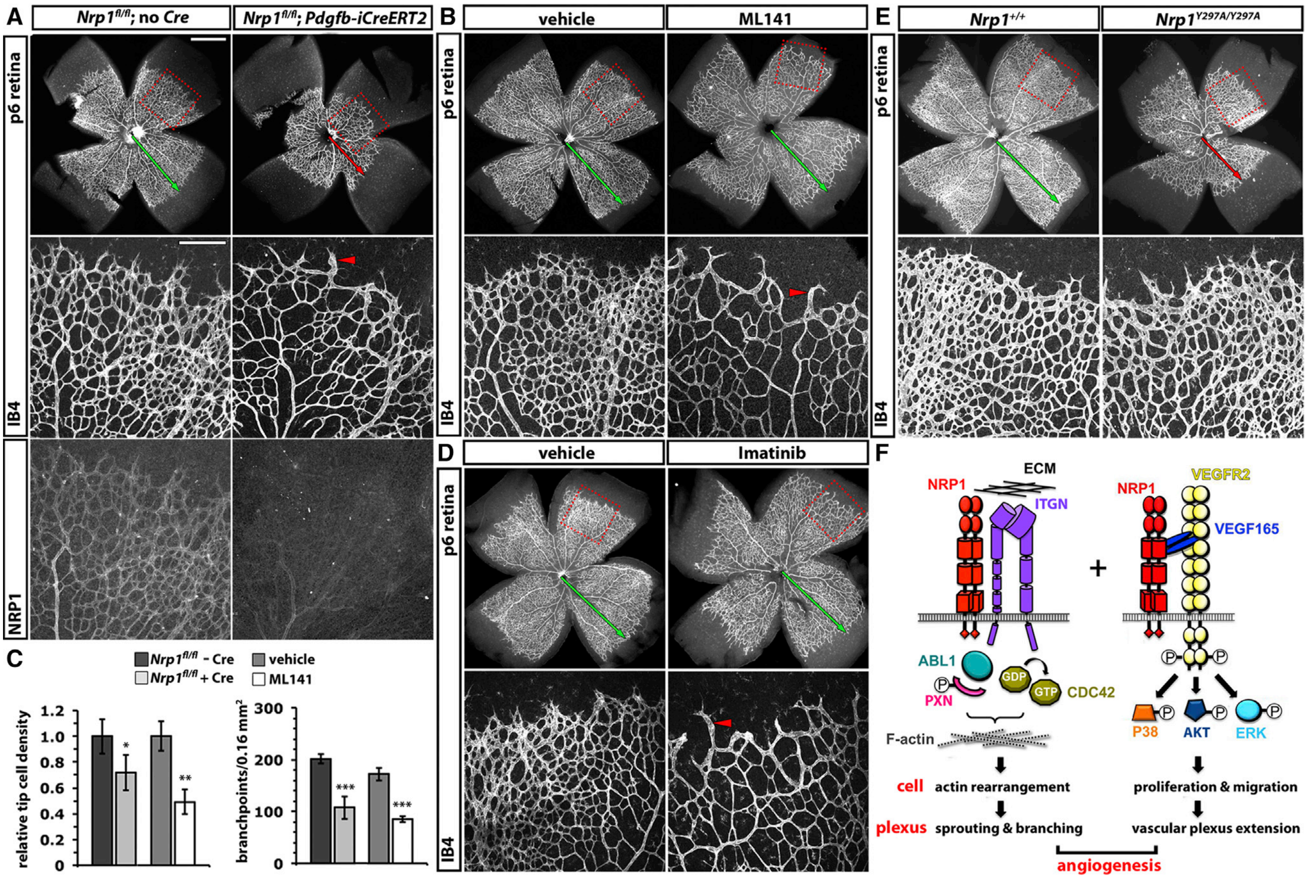

Figure 7. CDC42 Inhibition Impairs Retinal Vessel Sprouting and Branching Similarly to ABL Inhibition but Differently to Loss of NRP1-Dependent VEGF-A Signaling

(A–C) Comparison of retinal vascular defects after endothelial NRP1 loss or CDC42 inhibition. IB4 labeling of P6 retinal vasculature from littermate *Nrp1*$^{fl/fl}$ mice lacking *Cre* (n = 8) or expressing *Pdgfb-iCre-ERT2-Egfp* (n = 6) after daily tamoxifen injection from P2 to P5 (A) or from littermate wild-type mice treated daily from P2 to P5 with vehicle (n = 3) or ML141 (n = 4) (B). In (A), retinas were co-immunolabeled for NRP1 to demonstrate knockdown in mutants. (C) Quantification of filopodial bursts at the vascular front and branchpoints behind the vascular front; mean ± SD; asterisks indicate *$p < 0.05$, **$p < 0.01$, and ***$p < 0.001$.

(D and E) Comparison of retinal vascular defects after ABL kinase inhibition or loss of VEGF-A binding to NRP1. IB4 labeling of P6 retinal vasculature from littermate wild-type mice treated daily from P2 to P5 with vehicle or Imatinib (D) or *Nrp1*$^{Y297A/Y297A}$ mice lacking VEGF-A binding to NRP1 and wild-type littermates (E).

For (A), (B), (D), and (E), the top panels show low-magnification images of retinal flat mounts (scale bar, 1 mm) and boxed areas are shown at higher magnification below each panel (scale bar, 200 μm). The green arrow indicates normal vascular extension, the red arrows defective vascular extension, and the arrowheads abnormally long and wide sprouts without lateral protrusions or connections.

(F) Schematic representation of NRP1 roles in angiogenesis. NRP1 enables the ECM-dependent activation of ABL1 and CDC42 in addition to its classical role as a VEGFR2 co-receptor in VEGF-A signaling.

CDC42 Inhibition Impairs Retinal Angiogenesis Similarly to Loss of ECM Signaling through NRP1

To provide additional, genetic evidence for the role of NRP1 in CDC42 activation during angiogenesis, we also compared the vascular phenotypes of mice treated with ML141 or carrying a genetic mutation that disrupts endothelial NRP1 expression. For these experiments, we used the mouse retina as a model of NRP1-dependent angiogenesis (1) because it is accessible to small-molecule inhibitors and suited to the tamoxifen-inducible, endothelial specific deletion of floxed target genes and (2) because this model was recently used to establish distinct roles for ECM-induced and VEGF-A-induced NRP1 pathways in angiogenesis (Fantin et al., 2014; Gelfand et al., 2014; Raimondi et al., 2014). To obtain endothelial *Nrp1* mutants defective in both ECM and VEGF-A signaling, we used mice with conditional *Nrp1* null (floxed) alleles (*Nrp1*$^{fl/fl}$) expressing a tamoxifen-inducible *Cre* transgene under the control of the endothelial *Pdgfb* promoter (*Pdgfb-iCreER-Egfp*) or littermate controls lacking the *Cre* transgene (Fantin et al., 2013a; Raimondi et al., 2014). Tamoxifen treatment from perinatal day 2 (P2) to P5 efficiently knocked down NRP1 in the mutant compared to littermate control retina at P6 (Figure 7A). As previously shown (Raimondi et al., 2014), NRP1 knockdown reduced vascular network density, with sprouts at the vascular front appearing longer and larger and fewer lateral connections between neighboring vessels (Figure 7A, red arrowhead).

Similar to NRP1 knockdown, CDC42 inhibition with ML141 from P2 to P5 reduced vascular network density at P6, with sprouts at the vascular front appearing longer and larger and fewer lateral connections between neighboring vessels (Figure 7B, red arrowhead). Quantitative analysis demonstrated a significant reduction in tip cell density at the vascular front in both NRP1-targeted and ML141-treated P6 retinas (Figure 7C). The number of vascular branchpoints, formed through the fusion of newly formed vessel sprouts, was also severely reduced after both NRP1 targeting and ML141 treatment (Figure 7C). Accordingly, retinas of NRP1-ablated and ML141-treated mice had a similarly reduced vascular complexity. As previously shown (Raimondi et al., 2014), the inhibition of ABL kinases, which operate downstream of ECM-induced NRP1 signaling in retinal angiogenesis, caused sparser and longer sprouts at the vascular front and reduced vascular network complexity in the P6 retina (Figure 7D) similar to the pharmacological inhibition of CDC42 (Figure 7B). The observation that targeting NRP1, ABL kinases, or CDC42 similarly affects the filopodia-led processes of vascular sprouting and branching in the retina supports the idea that these genes operate in a shared angiogenic tip cell pathway.

Endothelial NRP1 loss also severely inhibited vascular extension (Figure 7A, red arrow) (Raimondi et al., 2014). This phenotype was not observed after CDC42 inhibition (Figure 7B, green arrow; mean distance from the optic nerve head to the vascular front relative to the retinal radius ± SD: vehicle 0.78 ± 0.05 versus ML141 0.81 ± 0.03; $n \geq 3$ each; $p > 0.05$). This finding suggests that NRP1 also has CDC42-independent roles in angiogenesis. In agreement, abolishing VEGF-A binding to NRP1 in *Nrp1*$^{Y297A/Y297A}$ mice (Fantin et al., 2014) recapitulated the vascular extension defect caused by endothelial NRP1 loss in the P6 retina (Figure 7E, red arrow). Vice versa, vascular branching, which was severely compromised by endothelial NRP1 loss, ABL inhibition or CDC42 inhibition, was only mildly affected in *Nrp1*$^{Y297A/Y297A}$ P6 retinas (Figure 7E). Taken together with our in vitro studies in HDMECs, we conclude that NRP1 has multiple roles in angiogenesis, which include the ECM-dependent activation of both ABL kinases and CDC42 in addition to NRP1's classical role as a VEGFR2 co-receptor in VEGF-A signaling (Figure 7F).

DISCUSSION

Angiogenic vessel growth depends on the formation of new sprouts composed of endothelial tip and stalk cells, followed by the fusion of nascent sprouts into perfused circuits. Previously, we demonstrated that NRP1 promotes the function of tip cells, even though we had not identified the specific molecular mechanism involved (Fantin et al., 2013a). Now, we show that NRP1 stimulates tip cell behavior by enhancing endothelial actin remodeling and filopodia extension, which agrees with the observed enrichment of NRP1 on tip cells and their filopodia (Fantin et al., 2013a). Filopodia are particularly important for directional cell migration through their roles in sensing chemotactic and haptotactic cues in the extracellular environment; they also act as anchorage points for ECM attachment, likely generating tension to pull cells forward as they become motile (De Smet et al., 2009). During retinal angiogenesis, vessel sprouts headed by filopodia-studded tip cells migrate toward astrocyte-localized VEGF-A in the retinal periphery (Gerhardt et al., 2003; Ruhrberg et al., 2002), with filopodia being guided by astrocyte-derived FN (Raimondi et al., 2014; Stenzel et al., 2011). The process of VEGF-A/ECM-driven radial migration is accompanied by lateral branching and sprout fusion to add perfused loops to the expanding vessel network. With its dual role in VEGF-A and ECM signaling, demonstrated clearly for retinal angiogenesis (Figure 7) (Fantin et al., 2014; Gelfand et al., 2014; Raimondi et al., 2014), NRP1 may therefore be exquisitely poised to help translate microenvironmental cues into regulated actin dynamics for EC migration during blood vessel sprouting. However, NRP1 clearly acts in concert with other guidance pathways, as sprouting angiogenesis is compromised, but not abolished, by NRP1 ablation. The finding that NRP1 mutants with impaired filopodia formation show delayed, but not absent, vessel migration (Figures 1, 2, 5, and 7) agrees with prior observations in zebrafish, which showed that vascular sprout extension, even though inefficient, can still take place when filopodia formation is inhibited (Phng et al., 2013).

Morphologically identifiable tip cells were rare in *Nrp1* null mouse hindbrains (Figure 2), raising the possibility that NRP1 either controls tip cell specification or enables the execution of the tip cell cytoskeletal program. We observed that NRP1 targeting did not reduce the expression of genes known to be upregulated in tip cells and commonly used as tip cell markers (Figure 3). NRP1 is therefore not required for the genetic specification of tip cells. Rather than decreasing the expression of tip cell genes, NRP1 loss increased the expression of these genes, including *Vegfr2* (Figure 3). In agreement with increased *Vegfr2* expression, we observed increased expression of *Dll4*, a tip cell gene that is induced by VEGF-A signaling through VEGFR2 during tip cell selection (Jakobsson et al., 2010), arguing against defective VEGFR2 signaling as the cause of vascular defects in *Nrp1* null hindbrains. Moreover, consistent with increased *Dll4* expression, the notch target genes *Hes1* and *Hey1* were upregulated (Figure 3). The observation that the network of known tip cell markers and their targets is principally intact and in fact upregulated rather than downregulated may be a reflection of the more immature vessel plexus in *Nrp1* null mutants (Figure 1). Nevertheless, the upregulation of the tip cell specification program is unable to compensate for the sprouting defect in *Nrp1* null mutants, arguing for a major morphological defect that prevents proper execution of tip cell behavior.

CDC42 is a key mediator of filopodia assembly and actin remodeling in many cell types (De Smet et al., 2009) and is activated downstream of VEGF-A signaling in cultured ECs (Lamalice et al., 2004). Experiments with the embryoid body model of vasculogenesis had suggested that CDC42 is essential for blood vessel assembly by vasculogenesis, which takes place prior to angiogenesis (Qi et al., 2011). However, the early embryonic lethality of both constitutive and endothelial-specific CDC42 knockout mice due to defective vasculogenesis (Chen et al., 2000; Jin et al., 2013) had previously precluded the investigation of CDC42 in tip cell function and therefore sprouting

angiogenesis in the mouse. We have circumvented this limitation by targeting CDC42 activation with the allosteric inhibitor. We observed that CDC42 inhibition impaired ECM-induced actin cytoskeleton remodeling and the extension of filopodia-like microspikes in human ECs in vitro as well as endothelial filopodia extension and vessel branching during angiogenesis in mice and fish in vivo (Figures 4, 5, 6, and 7). Similar phenotypes were observed after endothelial NRP1 knockdown, consistent with our finding that NRP1 is required for CDC42 activation in ECs (Figure 4).

Strikingly, CDC42 inhibition affected retinal angiogenesis similarly to inhibiting ABL kinases (Figure 7), which are activated in a NRP1-dependent fashion after stimulating ECs with FN. Thus, we have previously shown that ABL1 knockdown in primary human ECs or treatment of perinatal mice with the ABL kinase inhibitor Imatinib impaired ECM-induced and NRP1-dependent actin cytoskeleton remodeling, filopodia extension, and vessel branching in ECs (Raimondi et al., 2014). Interestingly, prior observations had suggested that ABL kinases function upstream of CDC42 in myeloid cells after lysophosphatidic acid stimulation (Baruzzi et al., 2010). In agreement, we found that ABL1 is also required for CDC42 activation in ECM-stimulated ECs (Figure 4). As ABL1 forms a complex with NRP1 (Raimondi et al., 2014), and NRP1 forms a complex with CDC42 (Figure 4G), it is likely that the NRP1/ABL1 complex has a direct role in CDC42 activation, perhaps by localizing CDC42 to sites of actin remodeling. Additionally, a small but significant decrease in NRP1 protein levels caused by ABL1 knockdown (Raimondi et al., 2014) may also contribute to reduced CDC42 activation. In either scenario, the surprising similarity of phenotypes caused by ABL or CDC42 inhibition distinguishes ECM-induced NRP1 signaling functionally from VEGF-A-induced NRP1 signaling, which instead appears to be more important for chemotactic guidance and vascular extension (Figure 7), likely by potentiating VEGFR2 signaling in both tip and stalk cells (e.g., Gerhardt et al., 2003).

In summary, our findings show that NRP1 enables actin remodeling and filopodia formation in endothelial tip cells via CDC42 to help convert proangiogenic ECM signals into tip cell responses for directional vessel sprouting and branching (summarized in Figure 7F). This model differs substantially from prior models, which suggested that NRP1 functions mainly as a co-receptor for VEGFR2, another known tip cell gene, to enable VEGF-A mediated chemotactic guidance. Thus, both NRP1 functions may cooperate to ensure that angiogenic growth factor guidance and ECM-stimulated migration are coordinated to ensure the ordered vascularization of developing organs (model in Figure 7F).

The similar angiogenesis defects caused by loss of NRP1 in genetically targeted mice and MO-treated fish, together with the similarity to defects induced by pharmacological CDC42 inhibition in both species and also in human endothelial cells, argue that the mechanism of NRP1-mediated CDC42 regulation is conserved across species. In addition to its prominent role in endothelial cells, NRP1 is also expressed in cell types as diverse as neurons, immune cells, and tumor cells. Future work should therefore investigate whether NRP1-dependent CDC42 activation contributes to physiological or pathological contexts beyond its essential role in developmental angiogenesis.

EXPERIMENTAL PROCEDURES

Mouse Strains

Animal procedures were performed in accordance with institutional and UK Home Office ethical guidelines. For more information, see the Supplemental Experimental Procedures.

Whole-Mount Immunolabeling, Imaging, and Quantitative Analysis of Mouse Hindbrains and Retinas

Mouse embryo hindbrains and postnatal retinas were immunolabeled as described previously (Fantin et al., 2013b; Pitulescu et al., 2010). For the 3D analysis of EC filopodia morphology with Imaris (BitPlane), IB4+ blood vessels and macrophages in high-resolution confocal z stacks were masked, but filopodia extending from vessels were excluded from the mask; filopodia were automatically tracked with the Imaris Filament Tracer module. For more information, see the Supplemental Experimental Procedures.

Gene Expression Analyses

Hindbrain mRNA was extracted using TRI reagent (Sigma-Aldrich) and cDNA prepared using Superscript III reverse transcriptase (Invitrogen) for qRT-PCR using SYBR Green (Applied Biosystems). For more information, see the Supplemental Experimental Procedures.

Zebrafish

MOs were injected into *Tg(fli1a:EGFP)^{y5}* (Lawson and Weinstein, 2002) or *Tg(kdrl:HsHRAS-mCherry)^{s896}* (Chi et al., 2008) zebrafish embryos at the one-cell stage. Clustal Omega (https://www.ebi.ac.uk/Tools/msa/clustalo/) was used to align nucleotide and amino acid sequences. For more information, see the Supplemental Experimental Procedures.

Cell Culture and Cell Imaging

HDMECs were cultured in MV2 media with supplements (Promocell) and transfected with SMARTpool siRNA targeting NRP1 or ABL1 (Dharmacon) or *Silencer* negative control siRNA (Applied Biosystems) using Lipofectamine RNAIMAX (Life Technologies). For more information, see the Supplemental Experimental Procedures.

CDC42 Pull-Down Assay, Immunoprecipitation, and Immunoblotting

GTP-bound CDC42 was isolated with glutathione agarose beads bound to the p21-binding domain of PAK1 (Millipore) or the CDC42-binding domain of WASP (Cytoskeleton) via a GST tag and identified by immunoblotting of eluted proteins with an antibody for CDC42 (Millipore). For more information, see the Supplemental Experimental Procedures.

Statistical Analysis

We calculated p values with a two-tailed unpaired Student's t test or one-way ANOVA followed by a Tukey post hoc test with Prism 5 (GraphPad Software) or Excel 12.2.6 (Microsoft Office); $p < 0.05$ was considered significant.

SUPPLEMENTAL INFORMATION

Supplemental Information includes Supplemental Experimental Procedures, three figures, and two movies and can be found with this article online at http://dx.doi.org/10.1016/j.celrep.2015.05.018.

AUTHOR CONTRIBUTIONS

A.F. performed and analyzed the retina and zebrafish experiments, A.L. and C. Raimondi performed and analyzed the cell culture experiments, G.G. performed zebrafish experiments, V.S. performed mouse genotyping and husbandry, I.Z. provided a vital reagent, and A.F. and C. Ruhrberg designed the study, performed hindbrain experiments, and prepared the manuscript. All authors have approved the manuscript.

ACKNOWLEDGMENTS

We thank Laura Denti and the Biological Resources Unit staff at the UCL Institute of Ophthalmology for help with mouse husbandry, the UCL zebrafish facility for fish maintenance, and Steve Wilson, Miguel Tillo, Karl Matter, Ceniz Zihni, Anissa Chikh, Matthew Golding, and Maddy Parsons for valuable advice. Grants from the Wellcome Trust (095623/Z/11/Z) and the British Heart Foundation (PG/10/86/28622 to C. Ruhrberg) and the Medical Research Council (MR/L003775/1 to G.G.) supported this study.

Received: December 12, 2014
Revised: March 23, 2015
Accepted: May 11, 2015
Published: June 4, 2015

REFERENCES

Baruzzi, A., Iacobucci, I., Soverini, S., Lowell, C.A., Martinelli, G., and Berton, G. (2010). c-Abl and Src-family kinases cross-talk in regulation of myeloid cell migration. FEBS Lett. *584*, 15–21.

Benard, V., Bohl, B.P., and Bokoch, G.M. (1999). Characterization of rac and cdc42 activation in chemoattractant-stimulated human neutrophils using a novel assay for active GTPases. J. Biol. Chem. *274*, 13198–13204.

Bovenkamp, D.E., Goishi, K., Bahary, N., Davidson, A.J., Zhou, Y., Becker, T., Becker, C.G., Zon, L.I., and Klagsbrun, M. (2004). Expression and mapping of duplicate neuropilin-1 and neuropilin-2 genes in developing zebrafish. Gene Expr. Patterns *4*, 361–370.

Chen, F., Ma, L., Parrini, M.C., Mao, X., Lopez, M., Wu, C., Marks, P.W., Davidson, L., Kwiatkowski, D.J., Kirchhausen, T., et al. (2000). Cdc42 is required for PIP(2)-induced actin polymerization and early development but not for cell viability. Curr. Biol. *10*, 758–765.

Chi, N.C., Shaw, R.M., De Val, S., Kang, G., Jan, L.Y., Black, B.L., and Stainier, D.Y. (2008). Foxn4 directly regulates tbx2b expression and atrioventricular canal formation. Genes Dev. *22*, 734–739.

De Smet, F., Segura, I., De Bock, K., Hohensinner, P.J., and Carmeliet, P. (2009). Mechanisms of vessel branching: filopodia on endothelial tip cells lead the way. Arterioscler. Thromb. Vasc. Biol. *29*, 639–649.

del Toro, R., Prahst, C., Mathivet, T., Siegfried, G., Kaminker, J.S., Larrivee, B., Breant, C., Duarte, A., Takakura, N., Fukamizu, A., et al. (2010). Identification and functional analysis of endothelial tip cell-enriched genes. Blood *116*, 4025–4033.

Fantin, A., Vieira, J.M., Gestri, G., Denti, L., Schwarz, Q., Prykhozhij, S., Peri, F., Wilson, S.W., and Ruhrberg, C. (2010). Tissue macrophages act as cellular chaperones for vascular anastomosis downstream of VEGF-mediated endothelial tip cell induction. Blood *116*, 829–840.

Fantin, A., Schwarz, Q., Davidson, K., Normando, E.M., Denti, L., and Ruhrberg, C. (2011). The cytoplasmic domain of neuropilin 1 is dispensable for angiogenesis, but promotes the spatial separation of retinal arteries and veins. Development *138*, 4185–4191.

Fantin, A., Vieira, J.M., Plein, A., Denti, L., Fruttiger, M., Pollard, J.W., and Ruhrberg, C. (2013a). NRP1 acts cell autonomously in endothelium to promote tip cell function during sprouting angiogenesis. Blood *121*, 2352–2362.

Fantin, A., Vieira, J.M., Plein, A., Maden, C.H., and Ruhrberg, C. (2013b). The embryonic mouse hindbrain as a qualitative and quantitative model for studying the molecular and cellular mechanisms of angiogenesis. Nat. Protoc. *8*, 418–429.

Fantin, A., Herzog, B., Mahmoud, M., Yamaji, M., Plein, A., Denti, L., Ruhrberg, C., and Zachary, I. (2014). Neuropilin 1 (NRP1) hypomorphism combined with defective VEGF-A binding reveals novel roles for NRP1 in developmental and pathological angiogenesis. Development *141*, 556–562.

Gelfand, M.V., Hagan, N., Tata, A., Oh, W.J., Lacoste, B., Kang, K.T., Kopycinska, J., Bischoff, J., Wang, J.H., and Gu, C. (2014). Neuropilin-1 functions as a VEGFR2 co-receptor to guide developmental angiogenesis independent of ligand binding. eLife *3*, e03720.

Gerhardt, H., Golding, M., Fruttiger, M., Ruhrberg, C., Lundkvist, A., Abramsson, A., Jeltsch, M., Mitchell, C., Alitalo, K., Shima, D., and Betsholtz, C. (2003). VEGF guides angiogenic sprouting utilizing endothelial tip cell filopodia. J. Cell Biol. *161*, 1163–1177.

Gerhardt, H., Ruhrberg, C., Abramsson, A., Fujisawa, H., Shima, D., and Betsholtz, C. (2004). Neuropilin-1 is required for endothelial tip cell guidance in the developing central nervous system. Dev. Dyn. *231*, 503–509.

Heasman, S.J., and Ridley, A.J. (2008). Mammalian Rho GTPases: new insights into their functions from in vivo studies. Nat. Rev. Mol. Cell Biol. *9*, 690–701.

Hellström, M., Phng, L.K., Hofmann, J.J., Wallgard, E., Coultas, L., Lindblom, P., Alva, J., Nilsson, A.K., Karlsson, L., Gaiano, N., et al. (2007). Dll4 signalling through Notch1 regulates formation of tip cells during angiogenesis. Nature *445*, 776–780.

Heyman, I., Kent, A., and Lumsden, A. (1993). Cellular morphology and extracellular space at rhombomere boundaries in the chick embryo hindbrain. Dev. Dyn. *198*, 241–253.

Heyman, I., Faissner, A., and Lumsden, A. (1995). Cell and matrix specialisations of rhombomere boundaries. Dev. Dyn. *204*, 301–315.

Hillman, R.T., Feng, B.Y., Ni, J., Woo, W.M., Milenkovic, L., Hayden Gephart, M.G., Teruel, M.N., Oro, A.E., Chen, J.K., and Scott, M.P. (2011). Neuropilins are positive regulators of Hedgehog signal transduction. Genes Dev. *25*, 2333–2346.

Hong, L., Kenney, S.R., Phillips, G.K., Simpson, D., Schroeder, C.E., Nöth, J., Romero, E., Swanson, S., Waller, A., Strouse, J.J., et al. (2013). Characterization of a Cdc42 protein inhibitor and its use as a molecular probe. J. Biol. Chem. *288*, 8531–8543.

Jakobsson, L., Franco, C.A., Bentley, K., Collins, R.T., Ponsioen, B., Aspalter, I.M., Rosewell, I., Busse, M., Thurston, G., Medvinsky, A., et al. (2010). Endothelial cells dynamically compete for the tip cell position during angiogenic sprouting. Nat. Cell Biol. *12*, 943–953.

Jin, Y., Liu, Y., Lin, Q., Li, J., Druso, J.E., Antonyak, M.A., Meininger, C.J., Zhang, S.L., Dostal, D.E., Guan, J.L., et al. (2013). Deletion of Cdc42 enhances ADAM17-mediated vascular endothelial growth factor receptor 2 shedding and impairs vascular endothelial cell survival and vasculogenesis. Mol. Cell. Biol. *33*, 4181–4197.

Kawasaki, T., Kitsukawa, T., Bekku, Y., Matsuda, Y., Sanbo, M., Yagi, T., and Fujisawa, H. (1999). A requirement for neuropilin-1 in embryonic vessel formation. Development *126*, 4895–4902.

Kok, F.O., Shin, M., Ni, C.W., Gupta, A., Grosse, A.S., van Impel, A., Kirchmaier, B.C., Peterson-Maduro, J., Kourkoulis, G., Male, I., et al. (2015). Reverse genetic screening reveals poor correlation between morpholino-induced and mutant phenotypes in zebrafish. Dev. Cell *32*, 97–108.

Kolluri, R., Tolias, K.F., Carpenter, C.L., Rosen, F.S., and Kirchhausen, T. (1996). Direct interaction of the Wiskott-Aldrich syndrome protein with the GTPase Cdc42. Proc. Natl. Acad. Sci. USA *93*, 5615–5618.

Lamalice, L., Houle, F., Jourdan, G., and Huot, J. (2004). Phosphorylation of tyrosine 1214 on VEGFR2 is required for VEGF-induced activation of Cdc42 upstream of SAPK2/p38. Oncogene *23*, 434–445.

Lanahan, A., Zhang, X., Fantin, A., Zhuang, Z., Rivera-Molina, F., Speichinger, K., Prahst, C., Zhang, J., Wang, Y., Davis, G., et al. (2013). The neuropilin 1 cytoplasmic domain is required for VEGF-A-dependent arteriogenesis. Dev. Cell *25*, 156–168.

Lawson, N.D., and Weinstein, B.M. (2002). In vivo imaging of embryonic vascular development using transgenic zebrafish. Dev. Biol. *248*, 307–318.

Lee, P., Goishi, K., Davidson, A.J., Mannix, R., Zon, L., and Klagsbrun, M. (2002). Neuropilin-1 is required for vascular development and is a mediator of VEGF-dependent angiogenesis in zebrafish. Proc. Natl. Acad. Sci. USA *99*, 10470–10475.

Lewis, J.M., Baskaran, R., Taagepera, S., Schwartz, M.A., and Wang, J.Y. (1996). Integrin regulation of c-Abl tyrosine kinase activity and cytoplasmic-nuclear transport. Proc. Natl. Acad. Sci. USA *93*, 15174–15179.

Lobov, I.B., Renard, R.A., Papadopoulos, N., Gale, N.W., Thurston, G., Yancopoulos, G.D., and Wiegand, S.J. (2007). Delta-like ligand 4 (Dll4) is induced by VEGF as a negative regulator of angiogenic sprouting. Proc. Natl. Acad. Sci. USA *104*, 3219–3224.

Martyn, U., and Schulte-Merker, S. (2004). Zebrafish neuropilins are differentially expressed and interact with vascular endothelial growth factor during embryonic vascular development. Dev. Dyn. *231*, 33–42.

Mattila, P.K., and Lappalainen, P. (2008). Filopodia: molecular architecture and cellular functions. Nat. Rev. Mol. Cell Biol. *9*, 446–454.

Paridaen, J.T., Janson, E., Utami, K.H., Pereboom, T.C., Essers, P.B., van Rooijen, C., Zivkovic, D., and MacInnes, A.W. (2011). The nucleolar GTP-binding proteins Gnl2 and nucleostemin are required for retinal neurogenesis in developing zebrafish. Dev. Biol. *355*, 286–301.

Phng, L.K., Potente, M., Leslie, J.D., Babbage, J., Nyqvist, D., Lobov, I., Ondr, J.K., Rao, S., Lang, R.A., Thurston, G., and Gerhardt, H. (2009). Nrarp coordinates endothelial Notch and Wnt signaling to control vessel density in angiogenesis. Dev. Cell *16*, 70–82.

Phng, L.K., Stanchi, F., and Gerhardt, H. (2013). Filopodia are dispensable for endothelial tip cell guidance. Development *140*, 4031–4040.

Pitulescu, M.E., Schmidt, I., Benedito, R., and Adams, R.H. (2010). Inducible gene targeting in the neonatal vasculature and analysis of retinal angiogenesis in mice. Nat. Protoc. *5*, 1518–1534.

Potente, M., Gerhardt, H., and Carmeliet, P. (2011). Basic and therapeutic aspects of angiogenesis. Cell *146*, 873–887.

Qi, Y., Liu, J., Wu, X., Brakebusch, C., Leitges, M., Han, Y., Corbett, S.A., Lowry, S.F., Graham, A.M., and Li, S. (2011). Cdc42 controls vascular network assembly through protein kinase Cι during embryonic vasculogenesis. Arterioscler. Thromb. Vasc. Biol. *31*, 1861–1870.

Raimondi, C., Fantin, A., Lampropoulou, A., Denti, L., Chikh, A., and Ruhrberg, C. (2014). Imatinib inhibits VEGF-independent angiogenesis by targeting neuropilin 1-dependent ABL1 activation in endothelial cells. J. Exp. Med. *211*, 1167–1183.

Ruhrberg, C., Gerhardt, H., Golding, M., Watson, R., Ioannidou, S., Fujisawa, H., Betsholtz, C., and Shima, D.T. (2002). Spatially restricted patterning cues provided by heparin-binding VEGF-A control blood vessel branching morphogenesis. Genes Dev. *16*, 2684–2698.

Soker, S., Miao, H.Q., Nomi, M., Takashima, S., and Klagsbrun, M. (2002). VEGF165 mediates formation of complexes containing VEGFR-2 and neuropilin-1 that enhance VEGF165-receptor binding. J. Cell. Biochem. *85*, 357–368.

Stenzel, D., Lundkvist, A., Sauvaget, D., Busse, M., Graupera, M., van der Flier, A., Wijelath, E.S., Murray, J., Sobel, M., Costell, M., et al. (2011). Integrin-dependent and -independent functions of astrocytic fibronectin in retinal angiogenesis. Development *138*, 4451–4463.

Ulrich, F., Ma, L.-H., Baker, R.G., and Torres-Vázquez, J. (2011). Neurovascular development in the embryonic zebrafish hindbrain. Dev. Biol. *357*, 134–151.

Yu, H.H., Houart, C., and Moens, C.B. (2004). Cloning and embryonic expression of zebrafish neuropilin genes. Gene Expr. Patterns *4*, 371–378.

Cell Reports

Supplemental Information

NRP1 Regulates CDC42 Activation to Promote Filopodia Formation in Endothelial Tip Cells

Alessandro Fantin, Anastasia Lampropoulou, Gaia Gestri, Claudio Raimondi, Valentina Senatore, Ian Zachary, and Christiana Ruhrberg

Supplemental Information

Supplemental figures and legends:

Figure S1, related to Fig. 1. **Reduced NRP1 levels in heterozygous *Nrp1*-null mutant hindbrains.** (**A-D**) Non-saturated confocal z-stacks of E11.5 mouse hindbrains of the indicated genotypes, immunofluorescently labelled for IB4 and NRP1. (**B',D'**) The single NRP1 channels shown in (**B,D**) were converted into a heat map to illustrate reduced pixel intensity in heterozygous mutants, whereby areas of high intensity are shown in red and areas of low intensity are shown in (blue); arrowheads indicate examples of tip cells. Scale bar: 50 μm. (**E)** Quantitation of NRP1 pixel intensity in heterozygous mutants relative to wild type littermates.

Figure S2, related to Fig. 5**. Normal ISV sprouting in control MO- and *nrp1b* MO-treated zebrafish embryos and demonstration that neural apoptosis or proliferation defects are not responsible for ISV defects in *nrp1a/b* MO-treated zebrafish.** (**A**) Confocal z-stacks of trunks from MO-treated 32 hpf *Tg(fli1a:EGFP)^{y5}* zebrafish that were immunostained for GFP after apoptosis detection via TUNEL; embryos had been treated with control MO, *nrp1a/b* MO or *nrp1a/b* MO together with *tp53* MO, as indicated. The GFP single channels are shown in greyscale in the upper panels. The boxed areas are shown at higher magnification in the bottom panels. The squared bracket indicates the position of the neural tube. Note increased apoptosis in the neural tube of *nrp1a/b* MO-treated embryos, but not in fish treated with the *nrp1a/b* MO together with the *tp53* MO. (**B**) Confocal z-stacks of trunks from 32 hpf *Tg(kdrl:HsHRAS-mCherry)^{s896}* zebrafish immunostained for mCherry and pHH3 after treatment with control, *nrp1b* or *nrp1a/b* MO. The mCherry single channel is shown in greyscale in the upper panels. The boxed areas are shown at higher magnification in the bottom panels. Arrowheads indicate examples of proliferating endothelial cells in ISV sprouts. Scale bars: 100 μm.

Figure S3, related to Fig. 5. **Nrp1 knockdown reduces the migration speed of ISV sprouts.** Live imaging of *Tg(fli1a:EGFP)^{y5}* zebrafish embryos during ISV sprouting at 32 hpf. (**A**) Series of laser confocal projections extracted from time-lapse videos (supplemental Movie 1 and 2) shown for the trunk of a control (left side panels) and *nrp1a/b* MO-microinjected (right side panels) zebrafish embryo and visualised with Imaris normal shading volume rendering; scale bar: 25 μm. (**B**) Quantitation of the migration speed of single ISV sprouts; mean±SD, n≥5 sprouts; *P* value (*<0.05).

Supplemental movies and legends:

Movie S1, related for Figure 5. **Migration speed of ISV sprouts in control embryos.** Live imaging of *Tg(fli1a:EGFP)^{y5}* zebrafish embryo trunks during ISV sprouting at 32 hpf.

Movie S2, , related for Figure 5**. Migration speed of ISV sprouts after Nrp1 knockdown.** Live imaging of *Tg(fli1a:EGFP)^{y5}* zebrafish embryo trunks during ISV sprouting at 32 hpf after *nrp1a/b* MO-microinjection.

Supplemental Experimental Procedures

Mouse strains Animal procedures were performed in accordance with institutional and UK Home Office guidelines. Mice were mated in the evening and the morning of vaginal plug formation was counted as embryonic (E) day 0.5. We used mice carrying a null mutation for *Nrp1* on a CD1 background (Kitsukawa et al., 1997) to circumvent the embryonic lethality of homozygous mutants by E10.5 that is observed in the C57/Bl6 background (Jones et al., 2008). We also used *Nrp1*^{Y297A} mice carrying a Y297A mutation that prevents VEGF-A binding to NRP1 (Fantin et al., 2014). For tamoxifen-induced, endothelial specific targeting of *Nrp1,* we used mice carrying floxed conditional *Nrp1* null alleles (*Nrp1*$^{fl/fl}$) together with *Pdgfb-iCre-ERT2-Egfp* with codon-improved *Cre* on a C57/Bl6 background (Fantin et al., 2013a). Tamoxifen (Sigma) was dissolved in peanut oil at 2 mg/ml, and 0.1 mg was administered on P2 and P3 via subcutaneous injections, followed by intraperitoneal injections on P4 and P5. ML141 (Sigma) was dissolved at 8 mM in DMSO and diluted to 4 mM in saline solution for injection of 0.08 mg into pups, whilst Imatinib (Cambridge Bioscience) was dissolved at 10 mM in water and diluted to 5 mM in saline solution for injection of 0.2 mg; both inhibitors were administered with a delivery regime similar to that used for tamoxifen treatment.

Wholemount immunolabelling, imaging and quantitative analysis of mouse hindbrains and retinas Mouse embryo hindbrains and postnatal retinas were immunolabelled as described (Fantin et al., 2013b; Pitulescu et al., 2010). To visualise blood vessels, we used biotinylated IB4 (cat. no. L2140, Sigma UK) followed by Alexa-conjugated streptavidin (Life Technologies). Samples were imaged with a LSM710 laser scanning confocal microscopes (Zeiss, Jena, Germany). For the 3-dimensional analysis of EC filopodia morphology with Imaris (BitPlane), IB4+ blood vessels and macrophages from high-resolution confocal z-stacks were masked, but filopodia extending from vessels were excluded from the mask; filopodia were automatically tracked with the Imaris Filament Tracer module. The endothelial tip cell number as well as filopodia number, length and thickness were determined in a minimum of two 0.05 mm^2 hindbrain regions, and the counts for each hindbrain averaged to yield a value for each hindbrain. In some experiments, rat anti-PECAM (cat. no. 553370, BD Bioscience) was detected with horseradish peroxidase (HRP)-conjugated rabbit anti-rat IgG (DAKO UK) and HRP-labelled samples were imaged with an MZ16 stereomicroscope (Leica) equipped with a ProgRes C14 camera (Jenoptics). The number of vascular branchpoints at E12.5 was determined in 3 randomly chosen 0.25 mm^2 regions in each hindbrain and then averaged. Tip cell density in the vascular front of the retina was quantified by normalising the number of filopodial bursts to the length of the vascular front, which was measured with Image J (http://imagej.nih.gov/ij/index.html). Vascular branchpoints in postnatal retinas were quantified using the Imaris Filament Tracer module in a minimum of three 0.16 mm^2 capillary areas behind the vascular front, and the counts for each retina averaged to yield a value for each retina. For all experiments, we calculated the mean of 3-12 independent samples. To evaluate NRP1 protein levels *in situ*, we combined IB4 staining with immunolabelling, using goat anti-rat NRP1 (cat. no. AF566, R&D Systems) followed by Cy3-conjugated rabbit anti-goat Fab fragment (Jackson Immuno) and acquired confocal z-stacks of the labelled tissue; we then rendered the single channel confocal z-stacks of NRP1 staining into heat-maps using the rainbow palette in Zeiss LSM Image Browser, with red, green and blue indicating high, medium and low pixel intensity, respectively. Images were processed with Photoshop CS4 (Adobe Inc.).

Gene expression analyses Hindbrain mRNA was extracted using TRI reagent (Sigma) and cDNA prepared using Superscript III reverse transcriptase (Invitrogen). For PCR amplification, we used SybrGreen (Applied Biosystems, USA) and the following oligonucleotide primers: *Nrp1* 5'-GAAGGTGAAATCGGAAAAGG-3' and 5'-GGTCTGTTGGTTTTGCACAG-3'; *Dll4* 5'-GCAGCTGTAAGGACCAGGAG-3' and 5'-ATTCGCAGGCATAACTGGAC-3'; *Ang2* 5'-CACAGCGAGCAGCTACAGTC-3' and 5'-ATAGCAACCGAGCTCTTGGA-3'; *Pecam* 5'-CGATGCGATGGTGTATAACG-3' and 5'-GTCACCTTGGGCTTGGATAC-3'; *Apln* 5'-GAGGAAATTTCGCAGACAGC-3' and 5'-GAGGAACTTGGTGGGTGAGA-3'; *Vegfr2* 5'-TCACCGAGAACAAGAACAAA-3' and 5'-TCCTATATCCTACAACCACAA-3'; *Actb* 5'-AAGGCCAACCGTGAAAAGAT-3' and 5'-GTGGTACGACCAGAGGCATAC-3'; *Hes1* 5'-TACCTGAAACACAGCAAAGC-3' and 5'-GGTAAACGAGCTTCATCTGC-3'; *Hey1* 5'-GGACGAGAATGGAAACTTGAG-3' and 5'-TTTCTAGCTTAGCAGATCCCT-3'; *Cdh5* 5'-GATGCAGATGACCCCACTGT-3' and 5'-AGGGCATCTTGTGTTTCCAC-3'. Amplicons were analysed with the quantitative 7500 Real-Time PCR System (Applied Biosystems) and SDS software v2.3 (Applied Biosystems) and DART-PCR software (Peirson et al., 2003).

Zebrafish experiments We injected *Tg(fli1a:EGFP)*^{y5} (Lawson and Weinstein, 2002) or *Tg(kdrl:HsHRAS-mCherry)*^{s896} (Chi et al., 2008) zebrafish embryos at the one cell stage with 0.6 pmol of the *nrp1a/b* translation blocking MO 5'-GAATCCTGGAGTTCGGAGTGCGGAA-3', previously published as a *nrp1a* MO (Hillman et al., 2011; Lee et al., 2002), 1.2 pmol of the *nrp1b* splicing blocking MO 5'-TATACCTGTACGGTGTATCTCATAG-3' (Wang et al., 2007) or 2.5 pmol of the standard control MO 5'-CCTCTTACCTCAGTTACAATTTATA-3' (Gene Tools). In some experiments, we co-injected 1 pmol of the translation blocking *tp53* MO 5'-GCGCCATTGCTTTGCAAGAATTG-3' (Chen et al., 2005) that inhibits apoptosis (e.g. Paridaen et al., 2011). Cell lysates from 32 hpf embryos were immunoblotted with a rabbit monoclonal antibody specific for the NRP1 cytoplasmic domain (cat no. 2621-1, Epitomics). Clustal Omega (https://www.ebi.ac.uk/Tools/msa/clustalo/) was used to align nucleotide and amino acid sequences of the two Nrp1 homologs with MO and antibody target sequences, respectively. In some experiments, embryos were treated with 25 or 75 µM ML141 or vehicle (DMSO) in the aquarium water from 8 to 32 hpf. To image vascular development, embryos were incubated at 28°C with phenylthiourea to prevent melanisation. 32 hpf embryos were then formaldehyde-fixed and immunostained with rabbit anti-GFP (cat. no. 598, MBL), chicken anti-GFP (cat. no. GFP-1020, Aves Labs) or rabbit anti-pHH3 (cat. no. 06-570, Millipore) and Alexa-conjugated goat anti-rabbit or anti-chicken antibodies (Life Technologies). Terminal deoxynucleotidyl transferase dUTP nick end labelling (TUNEL) was performed with the ApopTag kit according to the manufacturer's instructions (Millipore). 3-dimensional analysis of vessel sprouts and filopodia was carried out with Imaris as follows. Blood vessels and filopodia were tracked in high-resolution confocal z-stacks using Imaris Filament Tracer, while vessel sprout volume was measured using Imaris Surface rendering. We calculated the mean sprout values from 5-10 embryos per group in 2 independent experiments. For live imaging, 32 hpf embryos were anaesthetised in 0.01% tricaine and embedded in 1.5% low-melting point agarose. Time-lapse analysis was carried out on a Leica SPE confocal microscope for 4 hours by acquiring z-stacks of 15-20 confocal slices each every 10 minutes. The z-stacks were flattened by maximum projection and arranged in a time series using Imaris.

Cell culture and cell imaging HDMEC were cultured in MV2 media with supplements (Promocell, UK) and transfected with SMARTpool siRNA targeting NRP1 or ABL1 (Dharmacon, USA) or *Silencer*® negative control siRNA (Applied Biosystems, UK) using Lipofectamine RNAIMAX (Life Technologies). To evaluate the effect of NRP1 knockdown, HDMEC were serum-starved overnight, detached and plated on glass coverslips that had been coated overnight with 10 µg/ml FN (Sigma). In some experiments, untransfected cells were treated with vehicle (DMSO) or 7.5 µM ML141 before plating on FN; in other experiments, we additionally stimulated cells with 5 ng/ml VEGF165 for 15 min (R&D Systems). Stimulated cells were fixed, labelled with Alexa488-conjugated phalloidin and DAPI (Sigma) and then imaged on a Zeiss LSM700 confocal microscope.

CDC42 pull down assay, immunoprecipitation and immunoblotting To isolate GTP-bound CDC42, two different pull down assays were performed according to the manufacturer's instructions using glutathione agarose beads that were bound to either the p21-binding domain of PAK1 (Millipore) or the CDC42-binding domain of WASP (Cytoskeleton) via a GST tag. Immunoblotting of the precipitated proteins with an antibody specific for CDC42 (Millipore) identified GTP-bound CDC42. For this experiment, we serum-starved HDMEC overnight, detached and plated them on tissue culture plastic coated with 10 µg/ml FN for 30 minutes; in some experiments, we additionally stimulated for 15 min with 5 ng/ml VEGF165. In some experiments, HDMEC were transfected with siRNA targeting NRP1, ABL1 or control siRNA prior to plating on FN; in other experiments, cells were treated with vehicle or ML141, as described above. HDMEC were lysed according to the manufacturer's instructions and 300 µg protein was incubated with 10 µg beads at 4°C for 60 minutes. The bead supernatant was collected as the input control, while the bead pellet was washed and boiled for 5 minutes in 40 µl 2.5x Laemmli sample buffer to elute bound protein. CDC42 activation was calculated as the ratio between CDC42, detected by immunoblotting after pull down, and GST, detected by immunoblotting after pull down, or GAPDH, detected by immunoblotting in the input lysate (bead supernatant). For immunoprecipitation, HDMEC were lysed in 50 mM Tris pH 8.0 containing 50 mM KCl and 1% (v/v) Triton X-100 as well as protease inhibitor cocktail 2 and phosphatase inhibitor cocktail (Sigma) and incubated with goat anti-NRP1 (cat. no. sc-7239, Santa Cruz Biotechnology) or control goat IgG (Santa Cruz Biotechnology). For immunoblotting, heat-denatured samples were transferred to nitrocellulose membrane (Whatman, USA) after electrophoretic separation. We used the following antibodies for immunoblotting: mouse anti-CDC42 (cat. no. 17-441, Millipore), anti-GST (cat. no. G1160, Sigma), rabbit anti-NRP1 (cat. no. D62C6, Cell Signaling), anti-pCRKL (cat. no. 3181, Cell Signaling), and anti-GAPDH (cat. no. ab9485, Abcam) followed by appropriate HRP-conjugated secondary antibodies (Sigma).

Statistical analysis Error bars represent the standard deviation of the mean, unless otherwise stated in the figure legends. To determine if two data sets were significantly different, we calculated the *P*-value by performing a two-tailed unpaired Student's t-test; to compare more than two data sets, we additionally performed a one-way ANOVA followed by a Tukey post-hoc test; a P-value < 0.05 was considered significant. Statistical analyses were performed with Prism 5 (GraphPad Software) or Excel 12.2.6 (Microsoft Office).

Supplemental References

Chen, J., Ruan, H., Ng, S.M., Gao, C., Soo, H.M., Wu, W., Zhang, Z., Wen, Z., Lane, D.P., and Peng, J. (2005). Loss of function of def selectively up-regulates Delta113p53 expression to arrest expansion growth of digestive organs in zebrafish. Genes Dev *19*, 2900-2911.

Chi, N.C., Shaw, R.M., De Val, S., Kang, G., Jan, L.Y., Black, B.L., and Stainier, D.Y. (2008). Foxn4 directly regulates tbx2b expression and atrioventricular canal formation. Genes Dev *22*, 734-739.

Fantin, A., Herzog, B., Mahmoud, M., Yamaji, M., Plein, A., Denti, L., Ruhrberg, C., and Zachary, I. (2014). Neuropilin 1 (NRP1) hypomorphism combined with defective VEGF-A binding reveals novel roles for NRP1 in developmental and pathological angiogenesis. Development *141*, 556-562.

Fantin, A., Vieira, J.M., Plein, A., Denti, L., Fruttiger, M., Pollard, J.W., and Ruhrberg, C. (2013a). NRP1 acts cell autonomously in endothelium to promote tip cell function during sprouting angiogenesis. Blood *121*, 2352-2362.

Fantin, A., Vieira, J.M., Plein, A., Maden, C.H., and Ruhrberg, C. (2013b). The embryonic mouse hindbrain as a qualitative and quantitative model for studying the molecular and cellular mechanisms of angiogenesis. Nature Protocols *8*, 418-429.

Hillman, R.T., Feng, B.Y., Ni, J., Woo, W.M., Milenkovic, L., Hayden Gephart, M.G., Teruel, M.N., Oro, A.E., Chen, J.K., and Scott, M.P. (2011). Neuropilins are positive regulators of Hedgehog signal transduction. Genes & Development *25*, 2333-2346.

Jones, E.A., Yuan, L., Breant, C., Watts, R.J., and Eichmann, A. (2008). Separating genetic and hemodynamic defects in neuropilin 1 knockout embryos. Development *135*, 2479-2488.

Kitsukawa, T., Shimizu, M., Sanbo, M., Hirata, T., Taniguchi, M., Bekku, Y., Yagi, T., and Fujisawa, H. (1997). Neuropilin-semaphorin III/D-mediated chemorepulsive signals play a crucial role in peripheral nerve projection in mice. Neuron *19*, 995-1005.

Lawson, N.D., and Weinstein, B.M. (2002). In vivo imaging of embryonic vascular development using transgenic zebrafish. Dev Biol *248*, 307-318.

Lee, P., Goishi, K., Davidson, A.J., Mannix, R., Zon, L., and Klagsbrun, M. (2002). Neuropilin-1 is required for vascular development and is a mediator of VEGF-dependent angiogenesis in zebrafish. Proceedings of the National Academy of Sciences of the United States of America *99*, 10470-10475.

Paridaen, J.T., Janson, E., Utami, K.H., Pereboom, T.C., Essers, P.B., van Rooijen, C., Zivkovic, D., and MacInnes, A.W. (2011). The nucleolar GTP-binding proteins Gnl2 and nucleostemin are required for retinal neurogenesis in developing zebrafish. Dev Biol *355*, 286-301.

Peirson, S.N., Butler, J.N., and Foster, R.G. (2003). Experimental validation of novel and conventional approaches to quantitative real-time PCR data analysis. Nucleic Acids Res *31*, e73.

Pitulescu, M.E., Schmidt, I., Benedito, R., and Adams, R.H. (2010). Inducible gene targeting in the neonatal vasculature and analysis of retinal angiogenesis in mice. Nat Protoc *5*, 1518-1534.

Wang, L., Dutta, S.K., Kojima, T., Xu, X., Khosravi-Far, R., Ekker, S.C., and Mukhopadhyay, D. (2007). Neuropilin-1 modulates p53/caspases axis to promote endothelial cell survival. PLoS ONE *2*, e1161.

Figure S1

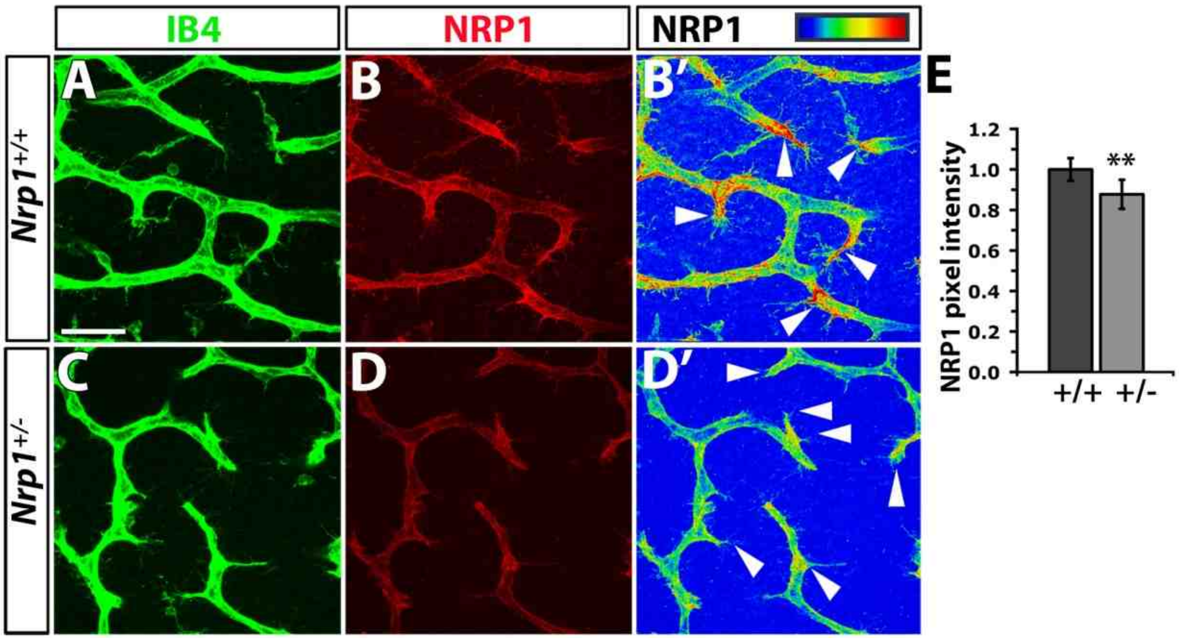

IB4
NRP1
NRP1
Nrp1+/+
Nrp1+/-
A
B
B'
C
D
D'
E
NRP1 pixel intensity
1.2
1.0
0.8
0.6
0.4
0.2
0.0
**
+/+
+/-

Figure S2

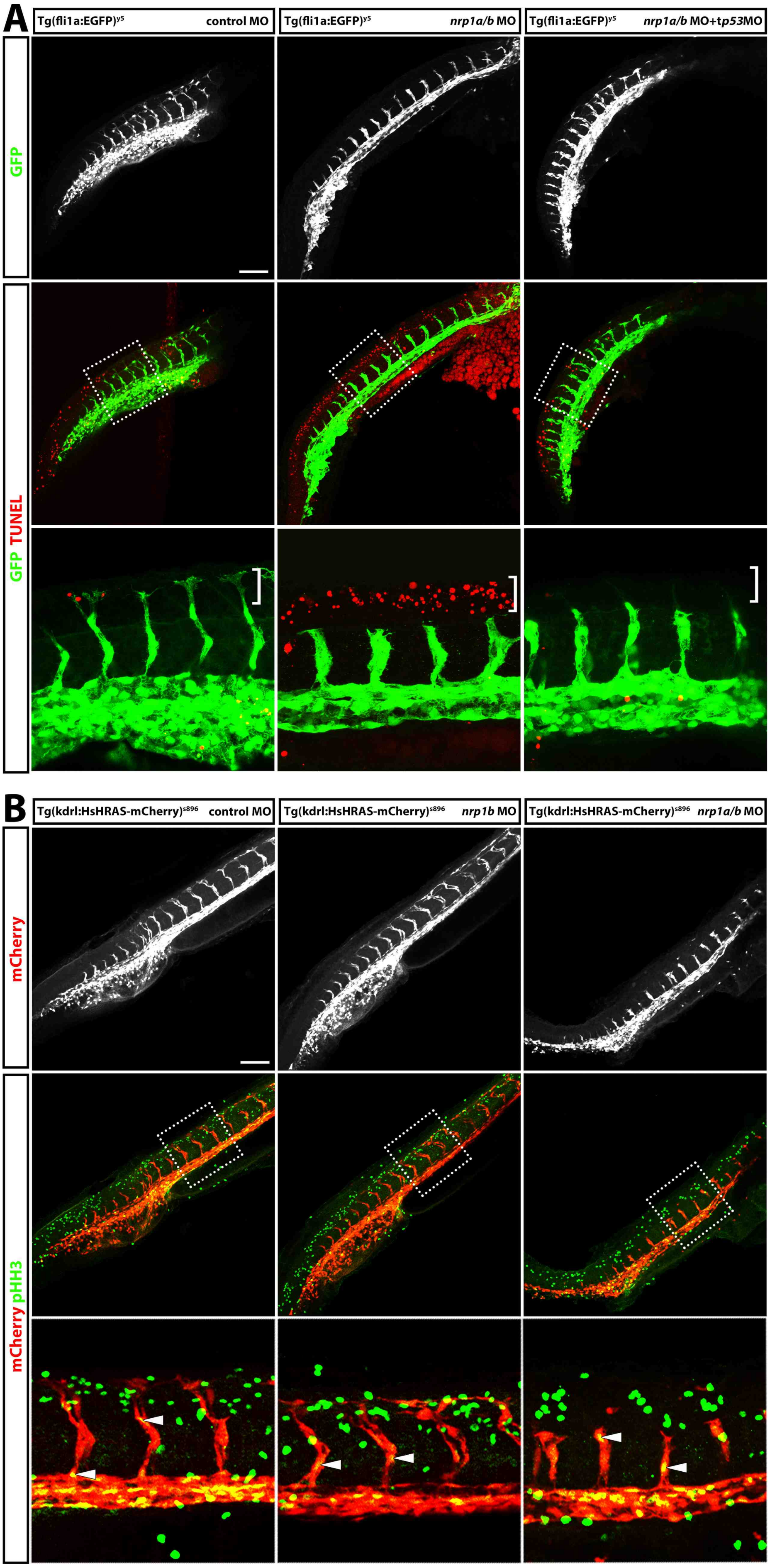
A
Tg(fli1a:EGFP)y5 control MO
Tg(fli1a:EGFP)y5 nrp1a/b MO
Tg(fli1a:EGFP)y5 nrp1a/b MO+tp53MO
GFP
GFP TUNEL
B
Tg(kdrl:HsHRAS-mCherry)s896 control MO
Tg(kdrl:HsHRAS-mCherry)s896 nrp1b MO
Tg(kdrl:HsHRAS-mCherry)s896 nrp1a/b MO
mCherry
mCherry pHH3

Figure S3

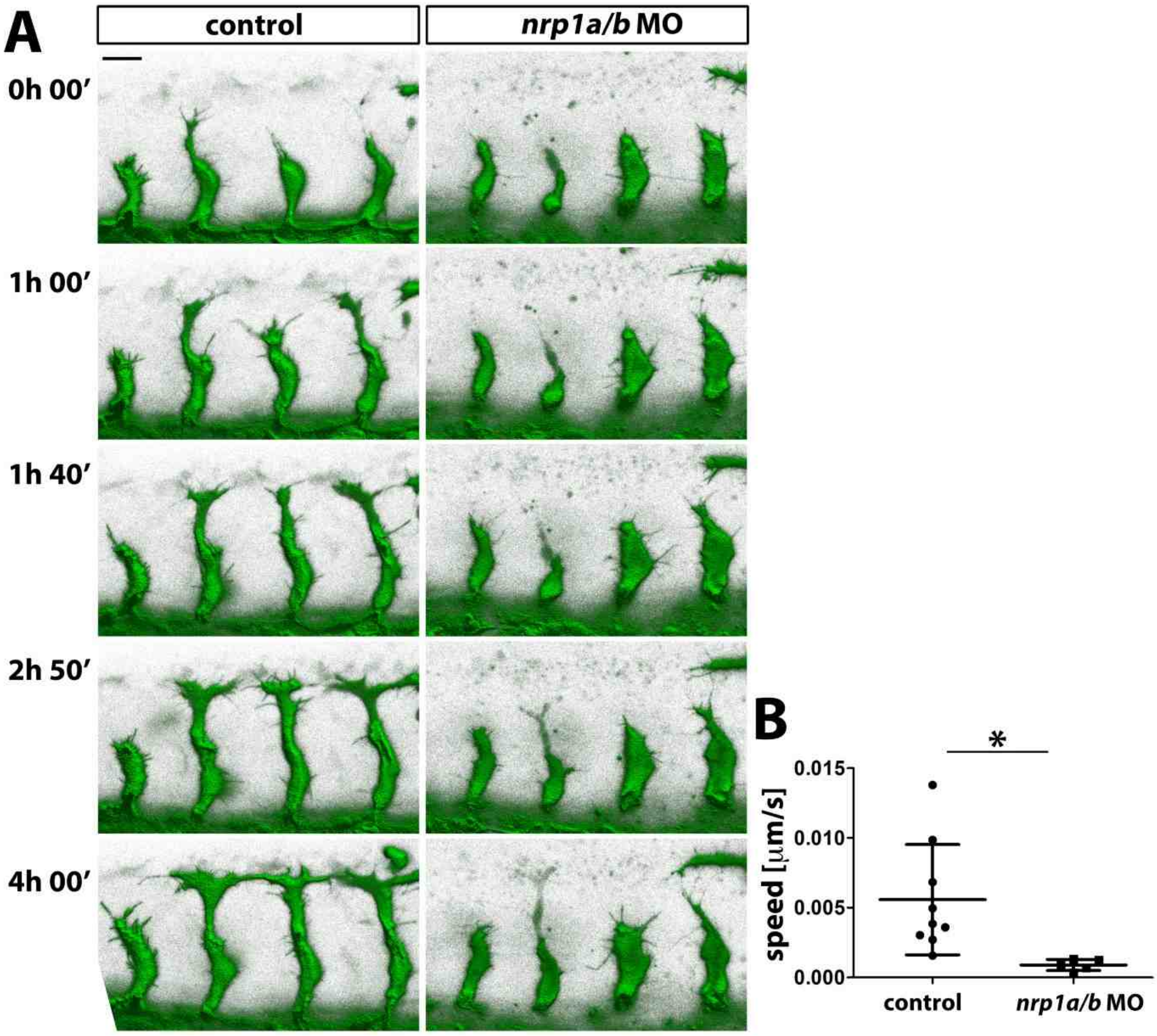
A
control
nrp1a/b MO
0h 00′
1h 00′
1h 40′
2h 50′
4h 00′
B
*
speed [μm/s]
0.015
0.010
0.005
0.000
control
nrp1a/b MO
